# Increased Hippocampal Excitability and Altered Learning Dynamics Mediate Cognitive Mapping Deficits in Human Aging

**DOI:** 10.1523/JNEUROSCI.0528-20.2021

**Published:** 2021-04-07

**Authors:** Nadine Diersch, Jose P. Valdes-Herrera, Claus Tempelmann, Thomas Wolbers

**Affiliations:** ^1^Aging and Cognition Research Group, German Center for Neurodegenerative Diseases (DZNE), Magdeburg 39120, Germany; ^2^Department of Neurology, Otto-von-Guericke University Magdeburg, Magdeburg 39120, Germany; ^3^Center for Behavioural Brain Sciences (CBBS), Otto-von-Guericke University Magdeburg, Magdeburg 39120, Germany

**Keywords:** aging, fMRI, learning, memory, spatial navigation, virtual reality

## Abstract

Learning the spatial layout of a novel environment is associated with dynamic activity changes in the hippocampus and in medial parietal areas. With advancing age, the ability to learn spatial environments deteriorates substantially but the underlying neural mechanisms are not well understood. Here, we report findings from a behavioral and a fMRI experiment where healthy human older and younger adults of either sex performed a spatial learning task in a photorealistic virtual environment (VE). We modeled individual learning states using a Bayesian state-space model and found that activity in retrosplenial cortex (RSC)/parieto-occipital sulcus (POS) and anterior hippocampus did not change systematically as a function learning in older compared with younger adults across repeated episodes in the environment. Moreover, effective connectivity analyses revealed that the age-related learning deficits were linked to an increase in hippocampal excitability. Together, these results provide novel insights into how human aging affects computations in the brain's navigation system, highlighting the critical role of the hippocampus.

**SIGNIFICANCE STATEMENT** Key structures of the brain's navigation circuit are particularly vulnerable to the deleterious consequences of aging, and declines in spatial navigation are among the earliest indicators for a progression from healthy aging to neurodegenerative diseases. Our study is among the first to provide a mechanistic account about how physiological changes in the aging brain affect the formation of spatial knowledge. We show that neural activity in the aging hippocampus and medial parietal areas is decoupled from individual learning states across repeated episodes in a novel spatial environment. Importantly, we find that increased excitability of the anterior hippocampus might constitute a potential neural mechanism for cognitive mapping deficits in old age.

## Introduction

Exploring our surroundings has always been one of the hallmarks of human identity. To do so, we need to rapidly generate spatial representations and flexibly retrieve them later. With advancing age, however, these abilities deteriorate considerably (Lester et al., 2017). Older adults are slower in learning novel environments and have problems to use this information later (Iaria et al., 2009). Moreover, learning landmark locations during exploratory navigation is more difficult for them (Yamamoto and DeGirolamo, 2012), whereas their spatial memory is relatively preserved for familiar environments (Rosenbaum et al., 2012). As a consequence, they may avoid unfamiliar places and become overwhelmed when confronted with changes in their environment.

Although core regions of the brain's navigation circuit in the medial temporal lobe are among the first to be affected during the progression from healthy aging to Alzheimer's disease (AD; Braak and Del Tredici, 2015), the neural mechanisms for age-related deficits in spatial learning are still poorly understood, even in healthy older adults. Studies in rodents and non-human primates showed that place cells in the CA3 subfield of the hippocampus exhibit higher firing rates in aged animals during navigation, and they fail to encode new information when rats encounter novel environments (Wilson et al., 2005; Thomé et al., 2016). Moreover, firing patterns of aged CA1 place cells are often unstable across repeated visits to the same environment (Barnes et al., 1997). In humans, in contrast, there is evidence for an age-related hypoactivation in the hippocampus and in medial parietal areas during spatial navigation (Moffat et al., 2006; Konishi et al., 2013).

However, whether activity changes in the aging brain are indicative of a compensatory mechanism or a correlate of aberrant processing is a long-standing issue in cognitive neuroscience research on aging (Grady, 2012; Morcom and Henson, 2018). Evidence from studies investigating age-related impairments in separating sensory input from mnemonic representations (i.e., pattern separation) suggests that hyperactivity in the dentate gyrus and CA3 may underlie memory deficits in healthy aging (Yassa et al., 2011; Reagh et al., 2018). Hippocampal hyperactivity has been further linked to preclinical markers for AD (Leal et al., 2017).

Age-related differences in neural activity may further depend on the point in time when activity is measured during task performance. Studies in younger adults showed that the engagement of the retrosplenial cortex (RSC) and the parieto-occipital sulcus (POS) together with the hippocampus changes over the course of learning (Wolbers and Büchel, 2005; Auger et al., 2015; Brodt et al., 2016; Patai et al., 2019). For example, Wolbers and Büchel (2005) showed that activity in the RSC/POS tracked the learning of relative landmark locations during spatial navigation and increased across learning sessions, whereas hippocampal activity reflected the amount of learning in a given session and decreased over time. Given the time course of its involvement during spatial learning, the RSC has been implicated in the retrieval of hippocampal-dependent memories. It receives inputs from CA1 and the subiculum (Kobayashi and Amaral, 2003; Bzdok et al., 2015) and is known to be involved in the integration of different spatial reference frames as well as in updating spatial representations (Epstein, 2008; Miller et al., 2014). The hippocampus, in turn, particularly its anterior portion, is known for its role in generating (spatial) representations (Zeidman and Maguire, 2016). Moreover, place-cell like activity in the RSC of mice relies on intact input from the hippocampus to support memory retrieval (Mao et al., 2018).

Here, we report findings from two experiments where we (1) characterized age-related problems in learning a novel environment; and (2) investigated the underlying neural mechanisms using fMRI. We focused on activity changes in the RSC/POS and the hippocampus and changes in effective connectivity within and between the two regions. This allowed us to test whether age-related problems in retrieving newly learnt information during spatial navigation is linked to a malfunctioning of the integration of hippocampal input within RSC/POS and/or a corrupted hippocampal signal.

## Material and Methods

### Participants

In the behavioral experiment, 17 younger (nine female, mean age: 24.0 ± 1.66, age range: 21–28) and 17 older adults took part (eight female, mean age: 66.4 ± 2.69, age range: 61–72). All of them were right-handed (LQ: 91.9 ± 11.0; Oldfield, 1971) and the older adults showed no signs of major cognitive impairment with scores higher than 23 in the Montreal Cognitive Assessment (MoCA score: 26.9 ± 2.18; Nasreddine et al., 2005; Luis et al., 2009).

To determine the required sample size for the fMRI experiment, we ran a power analysis with the effect size that was obtained in the behavioral experiment for the interaction between age group and learning blocks (η_p_^2^ = 0.188), using G*Power 3.1 (α = 0.05, 1-β = 0.95, two groups, eight repeated measurements; Faul et al., 2007). The power analysis further considered the most conservative correction for non-sphericity with 1/number of measurements – 1. This analysis indicated a requirement of 28 participants in total. We decided to double this number and recruited a total of 64 participants (27 younger adults, 37 older adults). Three participants (one younger and two older adults) were excluded from further analyses because they were identified as outliers in the fMRI data quality checks. In addition, one younger and three older adults were excluded because of problems in following task instructions and/or cybersickness. The final fMRI sample consisted of 25 younger (13 female, mean age: 23.4 ± 2.18, age range: 20–26) and 32 older adults (17 female, mean age: 67.3 ± 4.80, age range: 58–75). They were all right-handed (LQ: 90.4 ± 12.1; Oldfield, 1971) and the older adults did not show signs of major cognitive impairment (MoCA score: 27.6 ± 1.93, range: 25–31; Nasreddine et al., 2005).

Across experiments, participants had normal or corrected-to-normal vision and none of them reported a history of psychiatric or neurologic diseases or use of medication that might affect task performance or MRI scanning. In addition, most of the participants already participated in previous virtual reality (VR) experiments and, hence, were familiar with navigating in these kinds of setups. Participants provided informed consent and were paid for their participation in accordance with the local ethics committee.

### Virtual environment (VE)

**Figure 1.**
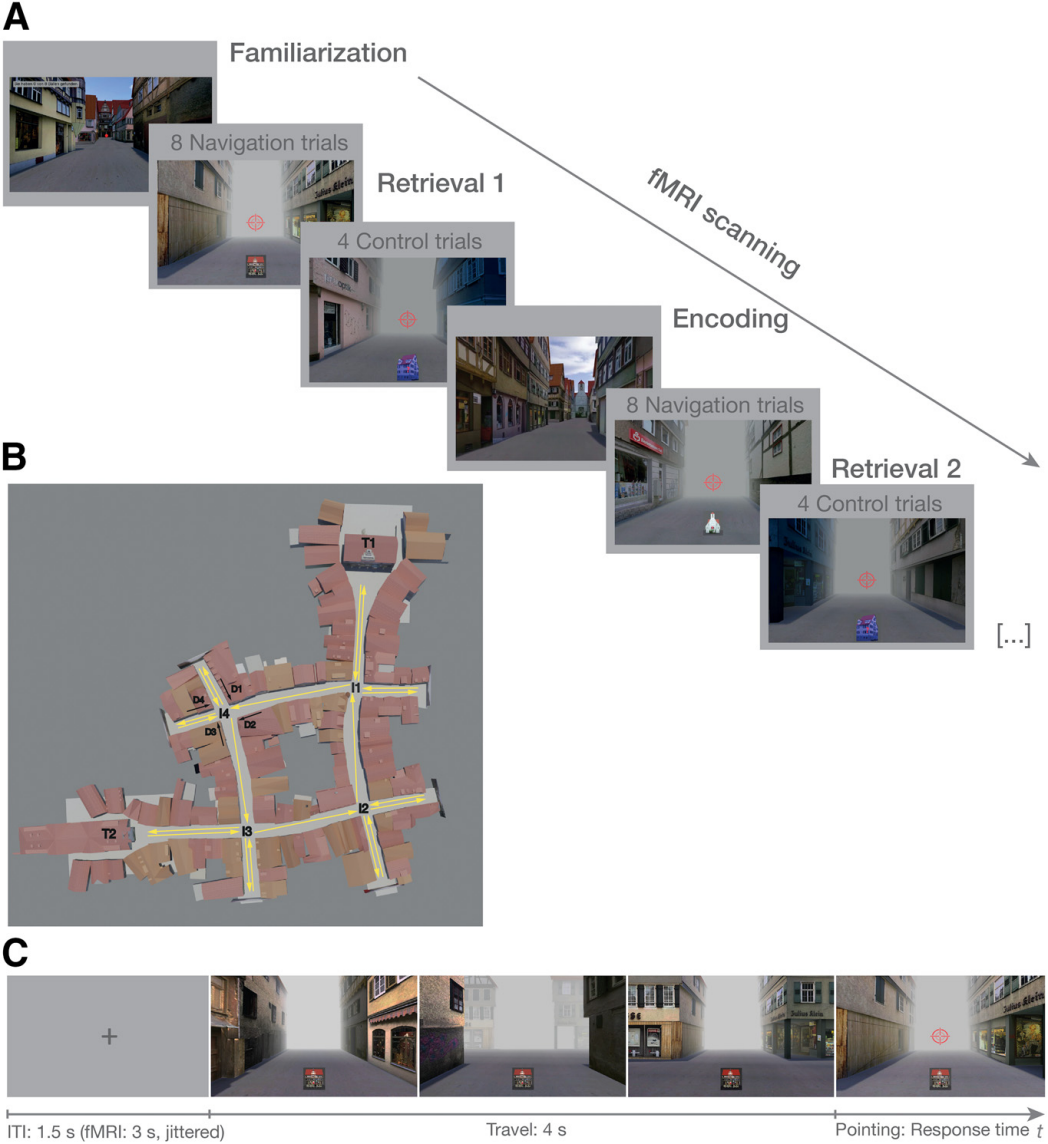
Spatial learning task. ***A***, Procedure of the fMRI experiment. After a familiarization phase outside of the scanner, eight retrieval phases, each comprising eight navigational retrieval trials and four control trials, alternated with seven encoding phases during scanning. In the behavioral experiment, the structure was the same except that 12 navigational retrieval trials per learning block were completed while the control trials were omitted. ***B***, Layout of the VE. The VE resembled a typical German historic city center and consisted of four interconnected intersections (I1–I4) that could be reached from four directions (D1–D4). At two intersections, a town hall (T1) and a church (T2) were placed at the end of one of the outgoing streets that served as target landmarks in the navigational retrieval trials. Yellow arrows exemplify one encoding tour that started from one of the target landmarks in clockwise or counterclockwise direction (a short segment of one tour is shown in Movie 1). ***C***, Structure of one example navigational retrieval trial to measure spatial learning. After fixation, participants were passively transported toward one of four intersections in the VE starting from one of the four streets leading toward that intersection (see Movie 2). Movement stopped at the center of the intersection, a red crosshair appeared, and participants were asked to move the crosshair in the direction of the respective target landmark. During the entire duration of the trial, a picture cue of the target landmark was displayed at the bottom of the screen, and the background was obscured by fog to prevent seeing the target landmarks. In the fMRI experiment, an additional jittered interval of 1 s (still phase) was added after the travel phase/before the crosshair appeared on screen.

Using 3ds Max (Autodesk), a novel VE was developed, which resembled a typical German historic city center consisting of town houses, shops and restaurants. The VE had a square-like spatial layout with four interconnected four-way intersections (Fig. 1*B*). At two intersections, a church and a town hall were placed at the end of one of the outgoing streets, whereas a 2D wall displaying a photograph texture of a street continuation bordered the remaining street ends. The VE was based on a 3D model of the old city center of Tübingen. All of the participants confirmed to have never visited Tübingen before the time of testing.

Movie 1. Exemplary segment of one of the tours through the VE in the encoding phases of the two experiments. 10.1523/JNEUROSCI.0528-20.2021.video.1

Movie 2. Example for a pointing trial in the retrieval phases of the two experiments. 10.1523/JNEUROSCI.0528-20.2021.video.2

### Experimental design and procedure

Vizard 5.0 (World Viz) was used to animate the experiments, which both started with a familiarization phase during which the participants encountered the VE for the first time. Their task during this phase was to collect tokens that were placed at the street ends by actively traveling the VE, using the four arrow keys of a standard computer keyboard. This phase ended once every token was collected, ensuring that they had visited every street at least once. It followed a short practice of the pointing task (eight trials) that was used to measure navigational retrieval in the experiments. In this way, the VE and the task were introduced in a step-wise manner to reduce the impact of different degrees of experience in handling VR setups on task performance (Diersch and Wolbers, 2019).

In the behavioral experiment, eight learning blocks were implemented during which eight retrieval phases alternated with seven encoding phases. One navigational retrieval phase consisted of 12 pointing trials. A pointing trial started with participants being passively transported toward one of the intersections starting from one of the four streets leading toward that intersection (Fig. 1*C*; see Movie 2 for an example trial). Duration of this travel phase was fixed to 4 s corresponding to 20 virtual meters. The movement stopped at the center of the intersection, a red crosshair appeared in the middle of the screen, and participants were asked to point in the direction of one of the two target landmarks. Pointing was performed by moving the crosshair to the left or right with the arrow keys of the keyboard. Once they believed to have reached the correct position, they confirmed their response by pressing the space bar. Participants were asked to respond as fast and accurately as possible with a time-out of 12 s (corresponding to 1½ 360° turns in the VE). The ITI (inter-trial interval), showing a fixation cross, was fixed to 1.5 s. Throughout each trial, a picture cue of the target landmark was displayed at the bottom of the screen and the background was obscured by fog to prevent participants from seeing the street ends or target landmarks during pointing. The first seven retrieval phases were followed by an encoding phase during which participants were passively transported around the whole VE (without fog), starting from one of the two target landmarks in clockwise or counterclockwise order, counterbalanced across the experiment (see Movie 1 for a short segment of one tour). During encoding, participants were instructed to pay close attention to the spatial layout of the VE and the location of the target landmarks. Passive transportation instead of self-controlled traveling was chosen to ensure that every participant experienced the VE for the same amount of time (duration: 2.88 min per tour). In total, participants performed 96 navigational retrieval trials (four intersections × four directions × two target landmarks × three repetitions) in a pseudo-randomized order, with the restriction that each intersection/target landmark combination was encountered starting from two of the four possible directions in the first half of the experiment. In the second half of the experiment, divided by a self-timed break, the respective other two directions were used, counterbalanced across participants. This allowed us to examine how experiencing familiar locations from a novel viewpoint affects pointing performance.

The fMRI experiment also consisted of eight learning blocks during which eight retrieval phases alternated with seven encoding phases (see Fig. 1*A* for the structure of the fMRI experiment). fMRI scanning started after a familiarization phase outside of the scanner with the same structure as in the behavioral experiment and a short practice phase during structural imaging. One retrieval phase consisted of eight navigational retrieval trials, which were followed by four control trials. These control trials also started with a 4-s travel phase toward an intersection, followed by a pointing phase with a crosshair on screen. Here, cued by a corresponding picture, however, participants were instructed to indicate which of the four corner buildings at the intersection had changed its color and was shaded in blue. Their responses in the control task were classified as correct if they were within ±25° from the middle of the respective building, approximately corresponding to its outline. Participants moved the crosshair with their index and middle finger for left and right turns and confirmed their responses with their right thumb on a five-key Lumitouch response box. Again, participants were asked to respond as fast and accurately as possible with a time-out of 12 s. The ITIs had a variable duration of 1–5 s with a mean of 3 s. During retrieval trials, an additional jittered interval of 0.5–1.5 s in duration with a mean of 1 s was included after the travel phase/before the crosshair appeared. The structure of the respective encoding tours was the same as in the behavioral experiment (passive traveling with a constant duration of 2.88 min per tour). In total, participants performed 64 navigational retrieval trials (four intersections × four directions × two target landmarks × two repetitions) without the change of directions from the first to the second half of the experiment as in the behavioral experiment. The change in directions was omitted in the fMRI environment to eliminate the potential influence of approaching the intersections from novel viewpoints and to accommodate a reduced number of trials because of the inclusion of the control task. In total, participants performed 32 control trials (four intersections × four directions × two repetitions). fMRI scanning consisted of three runs that were divided by short breaks with 24 navigational retrieval trials, 12 control trials and two encoding tours in the first run; 24 navigational retrieval trials, 12 control trials and three encoding tours in the second run; and 16 navigational retrieval trials, eight control trials, and two encoding tours in the third run.

### Bayesian modeling of performance data

**Figure 2.**
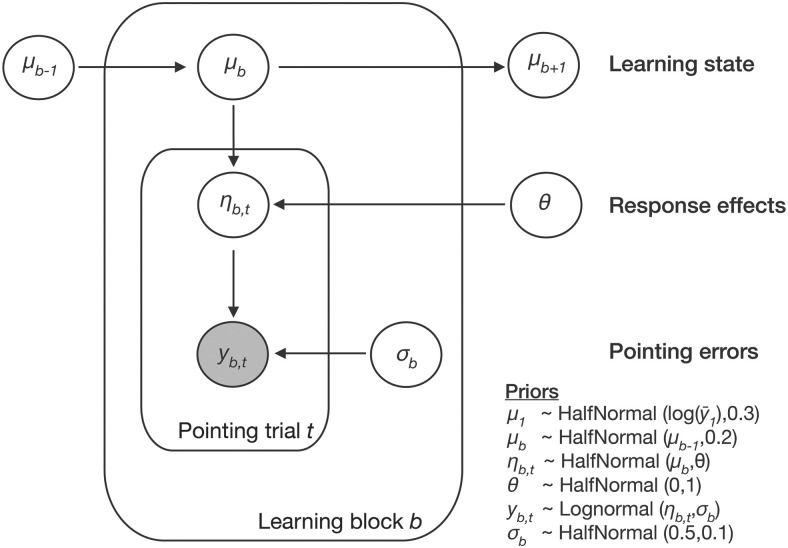
Bayesian state-space model to estimate the subject-specific hidden learning state per learning block (see Extended Data Figure 2-1 for the model code). Results of the posterior predictive checks of the model for representative individuals from each learning subgroup in the fMRI experiment and a histogram of the individuals' LOO differences for the comparison of the Bayesian state-space model to an alternative model that estimated the individuals' learning state trial-wise is depicted in Extended Data Figure 2-2.

In both experiments, subject-specific improvements in navigational performance were estimated by using a Bayesian implementation of a state-space model that is similar to a local level model where the trial outcomes, y, correspond to the observed level, and the state level represents the hidden learning state, μ (Fig. 2; Commandeur and Koopman, 2007). The hidden learning state, μ, is following a random walk such that the actual block learning state depends on the learning state from the previous block. Similar state-space models (Smith et al., 2007) have been used in previous studies to estimate spatial learning (Wolbers and Büchel, 2005; Auger et al., 2015). However, these studies modeled binary data on a trial-by-trial basis, whereas the present study used continuous performance outcomes and focused on estimating spatial learning block-wise instead of trial-wise. To model the learning state block-wise, an intermediate level accounts for the effects of the responses, η, and shrinks the effects of individual trials within a block toward the block-wise learning state. In this way, the model accounts for the fact that we can only measure behavioral performance but not the effect of learning or navigational improvement, which we expected to change from one encoding phase to the next but not necessarily from trial to trial. Introducing this intermediate level additionally allowed us to incorporate potential missing trials into the response effects, η. In case of missing trials, we estimated η ∼ HalfNormal (log($\bar{y}$*_b_*),1), i.e., using the log of the block mean as location parameter. The model was implemented using the Python interface to Stan, PyStan (Carpenter et al., 2017; Stan Development Team, 2017; see Extended Data Fig. 2-1 for the Stan code). To account for the substantial between-subject and within-subject variability of the data, weakly informative priors were chosen to provide vague guidance for effective sampling. The model was fit for each participant using four chains each with 4000 iterations, of which 2000 correspond to the warm up period, totaling 8000 postwarm-up draws. After inference, convergence of the chains was checked by means of the effective sample size and the potential scale reduction factor (Rhat), confirming that our chains mixed well (Gelman and Shirley, 2011).

10.1523/JNEUROSCI.0528-20.2021.f2-1 Extended Data Figure 2-1 Stan code of the Bayesian state-space model. Download Figure 2-1, DOCX file.

10.1523/JNEUROSCI.0528-20.2021.f2-2 Extended Data Figure 2-2 Results of the posterior predictive checks of the Bayesian state-space model for representative individuals from each learning subgroup (***A***, top learner young to ***E***, non-learner old; see Performance clustering section; the posterior predictive samples distribution, y_rep_, plotted together with the observed data points, y, per learning block) and (***F***) histogram of the individuals' LOO differences for the comparison of the Bayesian state-space model incorporating the effects of the responses, η, to an alternative model that estimated the individuals' learning state trial-wise. More positive values indicate a better fit of the first model. Download Figure 2-2, TIF file.

To determine the fit of our model to the data, we performed a posterior predictive check that compares the observed data with simulated data using samples from the posterior distribution. In Extended Data Fig. 2-2*A–E*, the posterior predictive samples distribution y_rep_ is plotted together with the observed data y for representative individuals from different learning subgroups in the fMRI experiment (see below, Performance clustering) showing that our model was adequate to capture the observed data. We further compared our model to an alternative, simpler model where η was removed (i.e., learning was estimated trial-wise instead of block-wise). Using a leave-one-out (LOO) cross-validation (Vehtari et al., 2017), point-wise out-of-sample prediction accuracies were estimated for both models. Comparing them confirmed that the model incorporating the intermediate layer accounting for the response effects, η, provided better fit to the data, as evidenced by positive LOO differences across the whole sample (sample mean = 1209, SEM = 242; see Extended Data Fig. 2-2*F* for a histogram showing the individual LOO difference values).

### fMRI acquisition parameters

Scanning was performed on a 3T Magnetom Prisma scanner (Siemens Healthcare) with a 20-channel head coil. High-resolution T1-weighted anatomic images were acquired using a MPRAGE sequence (1-mm isotropic resolution; TE = 2,82 ms; TR = 2500 ms; flip angle = 7°). In three functional runs, whole-brain T2*-weighted echo planar images with BOLD contrast were acquired in interleaved bottom-up order (36 slices, 3-mm isotropic resolution; TE = 30 ms; TR = 2000 ms; FoV = 216 mm; 72 × 72 image matrix; flip angle = 90°).

### Behavioral and fMRI statistical analyses

#### Behavioral analyses

Absolute pointing errors (i.e., the deviation of the subject's response from the direction toward the respective target landmark) served as performance measures in both experiments. In the behavioral experiment, we additionally analyzed response times given the change in directions from which the intersections were approached after the first half of the experiment. Where appropriate, ANOVAs were performed across learning blocks with age-group (younger adults, older adults) as between-subjects' variable. In a control analysis, we checked for potential biases in pointing behavior by applying circular statistics on the signed pointing error data relative to each target landmark for every intersection-direction combination, using the CircStat toolbox in MATLAB (Berens, 2009). In general, a threshold of *p* < 0.05 was considered significant (with correction for the number of tests where applicable).

#### Logistic regression model

With respect to the behavioral experiment, we were interested in whether two features that characterized age-related differences in performance could be used to predict the age group of our participants. The first feature was the mean amount of learning across all learning blocks, which was calculated based on the differences between individual learning state estimates, derived from the Bayesian state-space model, from two consecutive learning blocks. The estimates from the first learning block after the familiarization phase, during which participants encountered the VE for the first time, were subtracted from chance level performance (90°). In this way, learning-related improvements in performance were considered that already took place during the familiarization phase, resulting in pointing errors well below chance level in the first learning block for some participants. The second feature were the changes in response times after the directions changed from which the intersections were approached after the first half of the experiment. These two features were normalized and then fed into a logistic regression model as implemented in Scikit-learn (Pedregosa et al., 2011), with age group as target vector. The regularization parameter was set using a 10-fold nested cross-validation, and the performance of the model was assessed by computing the average area under the curve (AUC) for all folds. In this way, the probability of each individual belonging to the younger or the older age group could be estimated. The resulting probabilities are interpreted in terms of individual performance: those participants with a higher probability of belonging to the younger age group show better performance on the task while a higher probability of being in the older age group relates to poorer navigational performance.

#### Performance clustering

In the analysis of the behavioral data from the fMRI experiment, we assessed whether subjects could be clustered into different learning subgroups based on their performance. This allowed us to investigate learning-related differences in neural activity at the between-subjects level. For each participant, we created a distribution based on the difference of the latent state distributions of the last and first learning block to capture the overall amount of learning across the experiment. The mean and the SD parameters of this difference distribution were obtained by fitting it to a normal distribution using SciPy (Jones et al., 2001). In this way, the clustering provides a richer source of information to distinguish between different learning subgroups. For example, taking only the steepness of the curve across learning blocks into account, would not capture differences between very good learners, who learned most of the spatial layout already during familiarization, and very bad learners, with both groups exhibiting flat learning curves. However, they may differ in the uncertainty of their judgments, which is captured by the dispersion of the difference distribution. We used a K-means clustering algorithm as implemented in Scikit-learn (Pedregosa et al., 2011) to identify the centers of a predetermined number of clusters based on their distances to the data points. To obtain the optimal number of clusters to input into the K-means, we varied the number of possible clusters from three to seven and computed the mean Silhouette Coefficient of all samples per cluster as a measure for the distance between the resulting clusters with values ranging from −1 to 1 (negative values would indicate wrong cluster assignments and values near zero overlapping clusters). We found five to be the best choice for the number of learning subgroups in our sample (respective silhouette scores per tested cluster number: 3: 0.259, 4: 0.395, 5: 0.457, 6: 0.429, 7: 0.410). One should note that the results of this data-driven approach to characterize the heterogeneity within the two age groups are specific to our sample and cannot be generalized to the whole population. Different samples of younger and older adults might result in different learning clusters because of different performance levels.

#### fMRI image quality control and preprocessing

The imaging data were first transformed into the Brain Imaging Data Structure (BIDS) format (Gorgolewski et al., 2016). MRIQC (version 0.9.3; Esteban et al., 2017) was used for checking the quality of the MRI data. MRIQC utilizes tools from different software packages such as FSL or Advanced Normalization Tools (ANTs) to extract image quality metrics (IQMs) and generates visual reports at the individual and group level. This allows the evaluation of different characteristics of the structural and functional MRIs, for example, SNR/tSNR, sharpness, and presence of artifacts. Data from one younger adult and two older adults were consequently excluded from further analyses because of strong task-related movement and/or artifacts in several functional runs resulting in low-quality IQMs (e.g., high ghost-to-signal ratio, low tSNR). Next, motion correction, slice timing, co-registration, and normalization of the images was performed using fMRIprep version 1.0.0-rc5 (Esteban et al., 2019) that also draws on different software packages to provide the optimal implementation for different stages of preprocessing. For example, normalization to MNI space was performed using ANTs as a state-of-the-art medical image registration and segmentation toolkit. Finally, the data were smoothed with a 6 mm full-width at half maximum isotropic Gaussian kernel using SPM 12 (Wellcome Department of Imaging Neuroscience, London, United Kingdom).

#### Region of interest (ROI) definition

Based on results from previous studies (Wolbers and Büchel, 2005; Auger et al., 2015; Mao et al., 2018), we defined two ROIs, namely, the RSC/POS and the hippocampus. The single ROIs were created based on each participant's T1 structural scan using a semiautomated anatomic reconstruction and labeling procedure as implemented in FreeSurfer v6.0.0 (http://surfer.nmr.mgh.harvard.edu; Dale et al., 1999; Fischl et al., 1999). In each hemisphere, labels corresponding to the posterior-ventral part of the cingulate gyrus (area 10) and the POS (area 65) from the Destrieux Atlas and the hippocampus from the subcortical segmentation were extracted (Fischl et al., 2002; Destrieux et al., 2010). The two cortical labels were combined into one RSC/POS ROI. Each ROI was next transformed to MNI space. Hemispheres were combined to one bilateral ROI, thresholded at 0.5, and finally resampled to correspond to the resolution of our functional images. The ROIs were subsequently used in the univariate analysis and for the volumes of interest (VOIs) extraction in the effective connectivity analysis. In our definition of the hippocampus ROI, we did not separate between anterior and posterior hippocampus because previous fMRI studies have not reported a clear dissociation along the hippocampal long axis during navigation in healthy aging. Hence, strong a priori hypotheses about a potential anterior-posterior dissociation seemed unwarranted. Distinctions between anterior or posterior hippocampus in the results presentation refer to the location of the clusters we obtained in our analyses with foci at or anterior to y = −21 mm in MNI space being regarded as belonging to the anterior hippocampus (Poppenk et al., 2013).

#### fMRI univariate analysis

At the single-subject level, a general linear model (GLM) was specified with six regressors of interest for each learning block using a high-pass filter of 100 Hz. For the navigational as well as control retrieval trials, we created regressors for the 4 s travel phase and the pointing phase. For the encoding phases, regressors modeled periods when participants were located within 20 m of the intersection centers (corresponding to the area covered during the retrieval travel phases) as well as outside of these areas. Finally, the time of the button press was modeled as regressor of no interest. All regressors were convolved with the standard canonical hemodynamic response function (HRF) in SPM12. In addition, we included motion parameters, the frame-wise displacement (FD) and aCompCor values (Behzadi et al., 2007), as obtained from fMRIprep preprocessing, in the GLM to control for physiological and movement confounds. In aCompCor, significant principal components are derived from noise ROIs in which the time series data are unlikely to be modulated by neural activity. In this way, potential confounding effects of physiological fluctuations that may differ between age groups, such as cardiac pulsations and respiration-induced modulations, are removed from the fMRI time series.

We focused on interaction effects between conditions of interest and age group that are unlikely to be driven by group differences in neurovascular coupling, unlike main effects of age (Rugg and Morcom, 2005; see also Grinband et al., 2017). First, we contrasted navigational retrieval trials to control trials to identify general activation patterns in the RSC/POS and the hippocampus during spatial navigation in our complex real-world environment, similar to previous studies investigating age-group differences in spatial navigation (Moffat et al., 2006). We additionally contrasted the travel phases toward the intersections during navigational retrieval trials to the corresponding periods when participants encountered the same areas during the encoding tours. The within-subject effects of learning were assessed by using the normalized differences between learning state estimates (i.e., the outputs from the Bayesian state-space model) from consecutive learning blocks (i.e., amount of learning) as contrast weights over the regressors modeling each travel phase during navigational retrieval per learning block (compare Wolbers and Büchel, 2005). At the group level, the resulting individual contrast images were entered into two-sample *t* tests to assess interactions with age group. Finally, to check in which regions activity changes across learning blocks are modulated by the overall learning ability of the individual, we ran an additional analysis in which learning subgroup was added as covariate in the age-group comparisons at the second-level. All contrasts were evaluated at *p* < 0.001 (uncorrected) and we report activations that survived the familywise error (FEW)-correction for multiple comparisons using a threshold of *p* < 0.05 at the cluster level.

In a control analysis, we checked whether learning-related changes within the two ROIs could alternatively be driven by spatial computations in which older and younger adults engage in differently over the course of the experiment. We fitted a separate finite impulse response (FIR) GLM for each participant with the same regressors of interest as in our main GLM described above. The FIR model was set up with eight time bins (2-s duration each, total time window: 16 s) as a basis function for the HRF, and FIR time courses (percent signal change per time bin) were extracted within both the hippocampus and the RSC/POS ROIs for the regressors modeling the eight travel phases using MarsBar (Brett et al., 2002). For each participant, we then determined the time bin when the HRF reached its peak, separately for the beginning of learning (first four learning blocks) and the end of learning (last four learning blocks). This approach allowed us to test (1) whether the HRF reached its peak at different time points in the first versus the second half of the experiment, and (2) whether this time-to-peak differed between age groups.

#### Effective connectivity analysis

Effective connectivity within and between the hippocampus and the POS was examined using the parametric empirical Bayesian (PEB) approach in the context of dynamic causal modeling (DCM) as implemented in SPM12 (Friston et al., 2016).

##### GLM and VOI selection

For the DCM analysis, we created a GLM in which the time series from our three functional runs were concatenated and added regressors that modeled the mean signal for each run. The amount of learning per learning block was included as parametric modulation of the regressor modeling the travel phase during navigational retrieval trials for each participant. All other regressors were the same as in the first GLM although they were not modeled separately for each learning block. The sanity check of the concatenated GLM revealed that activity in the right anterior hippocampus (27, −9, −15, Z = 3.97; 27 voxels) decreased and activity in the left POS (−12, −63, 31, Z = 3.56; 42 voxels) increased with the amount of learning in younger adults (*p* < 0.05, FWE-corrected for the respective ROI). No additional activations emerged elsewhere in the brain. When testing for interactions between learning-related activity changes and age group within our ROIs, one cluster within bilateral POS extending to RSC was revealed (15, −66, 44, Z = 4.23; 12, −57, 4, Z = 4.21; −6, −66, 24, Z = 3.94; −15, −60, 28, Z = 3.61; 369 voxels). Thus, activity in this region increased with learning in younger adults but less so in older adults. The slight differences of these results to the ones from the first GLM are likely related to differences in the design of the two GLMs. Whereas the first GLM was optimized to capture our experimental design as precisely as possible by modeling all regressors of interest separately for each learning block, the concatenated GLM was optimized for the DCM analysis that relies on single-run time series.

BOLD time series were extracted for each individual using a t-contrast over the regressors modeling the travel phase during navigational retrieval and the amount of learning with a liberal threshold of *p* < 0.1 (Note that this threshold was only used for VOI selection, but not in the final DCM statistics). The principal eigenvariate was extracted around the group peak coordinates within the hippocampus and POS as obtained in the univariate analysis of the concatenated GLM and was allowed to vary as an 8-mm sphere centered on the subject-specific maximum constrained by a 24 mm sphere centered on the group maximum and the respective ROI mask. In this way, variation between individuals in the exact location of the effect was considered, given the high heterogeneity in our sample and slightly different peak voxels in the two GLMs. The extractions were corrected using an F-contrast that retained the effects of interest (navigational as well as control retrieval phases, encoding phases, button press) while partitioning out task-unrelated variance caused by head motion, for example. For participants for which no supra-threshold voxels were identified (three younger adults and one older adult), the threshold was lowered to *p* < 0.5 to extract BOLD time series (compare Zeidman et al., 2019b).

##### First-level DCM specification

We specified a bilinear, one-state DCM for each participant by setting the regressor modeling the travel phase during navigational retrieval trials as driving input entering the cortical network via the POS. The amount of learning per learning block was included as modulatory input on the bidirectional connections between hippocampus and POS. All inputs were mean-centered so that the A-matrix of the DCM represents the average connectivity across experimental conditions. We used stochastic DCM that seeks to improve model estimation by modeling random fluctuations and hidden neuronal causes in the differential equations of the neuronal states (Li et al., 2011; Daunizeau et al., 2012). In this way, the impact of potential confounding effects of variations in BOLD response caused by age is reduced. Bayesian group inversion was performed, providing estimates of the connection strength parameters that best explained the observed data per participant. Critically, within DCM PEB, at each iteration of the within-subject inversion, the individual priors are updated using the group average connection strengths as priors. Inspection of the single DCMs after inversion confirmed that our full model provided good fit to the observed data with an average of 44.5 ± 3.22% variance explained.

##### Second-level PEB model

Next, we created a second level PEB model over the parameters that included the group mean and age group as covariates to identify differences between younger and older adults. We further included learning subgroup and its interaction with age group as covariates in the model. The interaction term was modeled as the two main effects of age and learning group element-wise multiplied with the main effects being mean-centered and coded in a way that low/negative values represent younger or better performing individuals. A search over nested PEB models was performed by using Bayesian model comparison (BMC) that explores a space of models under the assumption that different combinations of the connections may exist across participants (Zeidman et al., 2019a). To search over hundreds of nested models incorporating different combinations of connections and group differences, Bayesian model reduction (BMR) was used that iteratively prunes parameters from the full model until model-evidence decreases. To reduce dilution of evidence, we separately checked for group differences in the A-matrix (average connectivity across experimental conditions) and the B-matrix (within-subject modulatory input of the amount of learning per block). We further performed a LOO cross-validation to check whether the model parameter that differed between older and younger adults could be used to predict the participants' age group.

### Data availability

Source data files for the main results figures and tables are stored at https://osf.io/fjbxu/. We additionally provide a key resources table listing all the software packages that were used in the current study. The Stan code of the Bayesian state-space model can be found in Extended Data Fig. 2-1.

## Results

Findings are reported from two separate samples comprising healthy younger and older adults who performed a spatial learning task either purely behaviorally (17 younger adults and 17 older adults) or in a combined fMRI-behavioral experiment (25 younger adults and 32 older adults). In both experiments, following an initial familiarization phase before testing/outside of the scanner, eight learning blocks were implemented during which eight retrieval phases alternated with seven encoding phases. We used the angular deviation of the participants' response from the respective target landmark (i.e., absolute pointing errors) to measure performance improvements across learning blocks. However, performance in these kinds of tasks can be corrupted by various noise sources and, hence, might not accurately reflect the actual learning state of the participant. Therefore, subject-specific improvements in navigational performance were estimated by using a Bayesian implementation of a state-space model that disambiguated learning from random trial-by-trial fluctuations in performance. We used the outputs of the model in the analysis of the fMRI data to examine intraindividual and interindividual differences in learning.

### Behavioral experiment

#### Lower performance and reduced learning in older adults

**Figure 3.**
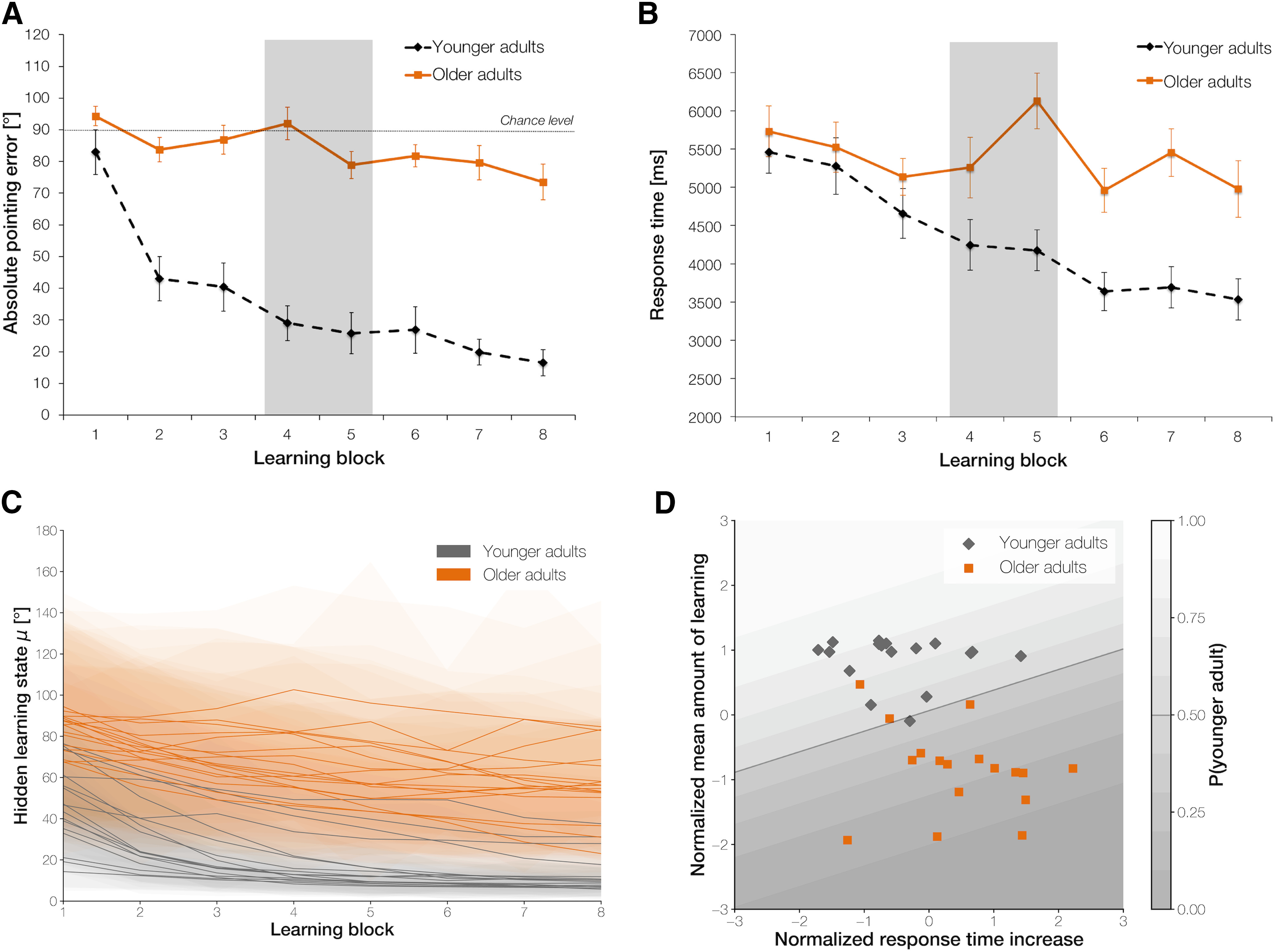
Performance data in the behavioral experiment. ***A***, Average absolute pointing errors and (***B***) response times across the eight learning blocks in older (solid line) and younger adults (dashed line; highlighted in gray is the fourth and fifth learning block where the change in directions took place from which the intersections were approached). Error bars denote SEM. See also Extended Data Figure 3-1*A*,*B* for average pointing errors per learning block for each participant in each age group. ***C***, Mean estimated performance improvement (hidden learning state) of each participant in the older (orange) and younger (gray) age group, including the SD of the posterior distributions (shaded area) across learning blocks. ***D***, Logistic regression results classifying age group membership based on two behavioral performance features, i.e., the mean amount of learning across the experiment and the increase in response times after the first half of the experiment. Shaded lines depict the probability of being classified as a younger adult.

An ANOVA with learning block (1–8) as repeated measures variable and age group (younger adults, older adults) as between-subjects variable on the average absolute pointing errors showed significant main effects of learning block, *F*_(7,224)_ = 19.5, *p* < 0.001, η_p_^2^ = 0.379, and age group, *F*_(1,32)_ = 85.2, *p* < 0.001, η_p_^2^ = 0.727. This was modulated by a significant interaction between the two factors, *F*_(7,224)_ = 7.40, *p* < 0.001, η_p_^2^ = 0.188. At the beginning, both age groups performed around chance level (90°), although older adults had spent significantly more time than younger adults (M_old_ = 534 ± 161 s; M_young_ = 218 ± 41.4 s; *t*_(16.9)_ = −7.63, *p* < 0.001, *d* = 2.69) in the initial familiarization phase of the experiment, during which they encountered the VE for the first time. Over the course of the experiment, however, older adults showed lower performance and less improvement compared with younger adults (Fig. 3*A*). The change in direction from the first to the second half of the experiment did not have a major effect on this pattern of results as implied by a non-significant interaction between learning block and age group when directly comparing the fourth and fifth learning block, *F*_(1,32)_ = 1.96, *p* = 0.171, η_p_^2^ = 0.058. A separate ANOVA within the older age group on pointing performance per learning block confirmed that older adults generally improved on the task over time as evidenced by a significant main effect of learning, *F*_(7,112)_ = 2.58, *p* = 0.017, η_p_^2^ = 0.139. According to the outputs of the Bayesian state-space model (Fig. 3*C*; see Extended Data Fig. 3-1*A*,*B* for average pointing errors per learning block for each participant), most of the younger adults learned the spatial layout of the VE very fast, reaching ceiling performance already after the first few learning blocks. The older adults, in contrast, differed more widely in their ability to learn.

10.1523/JNEUROSCI.0528-20.2021.f3-1 Extended Data Figure 3-1 Average absolute pointing errors per learning block for each participant in (***A***) the younger and (***B***) the older age group in the behavioral experiment and for each participant in (***C***) the younger and (***D***) the older age group in the fMRI experiment. Download Figure 3-1, TIF file.

To investigate potential biases in pointing behavior that might differ between the age groups, such as an increased tendency to point along streets, circular statistics were applied on the signed pointing error data relative to each target landmark for every intersection-direction combination. From the 32 age-group comparisons (four intersections × four directions × two target landmarks), only seven reached significance as determined by a Watson–Williams test, all *p* ≤ 0.047. Older adults showed larger deviation from the correct angle than younger adults in six of the seven instances. The direction of the deviations in pointing (e.g., to the left or right relative to the target landmark), however, varied, and none of the effects survived when correcting for multiple comparisons.

#### Higher uncertainty when viewpoints are changing in older adults

An ANOVA with learning block (1–8) as repeated measures variable and age group (younger adults, older adults) as between-subjects variable on the response time data confirmed significant main effects of learning block, *F*_(7,224)_ = 9.26, *p* < 0.001, η_p_^2^ = 0.225, and age group, *F*_(1,32)_ = 10.5, *p* = 0.003, η_p_^2^ = 0.247. Compared with older adults, younger adults responded quicker and showed a steeper decline in response times over time as revealed by a significant interaction between learning block and age group, *F*_(7,224)_ = 4.29, *p* = 0.001, η_p_^2^ = 0.118. Notably, when comparing the fourth and fifth learning blocks, a significant interaction between learning block and age group was obtained, *F*_(1,32)_ = 9.34, *p* = 0.004, η_p_^2^ = 0.226. Older but not younger adults showed a substantial increase in response times in the fifth learning block when the intersections were encountered from novel directions (Fig. 3*B*). This result cannot be explained by a confound between pointing performance and required turning at the intersections because the required amount of turning to perform accurately on the task varied from trial to trial, depending on the specific intersection-direction-target landmark combination. Moreover, it was kept constant across experiment halves and participants (average turning direction: 135°). When considering the fourth and fifth block only, the correct turning angle did not differ between blocks, age groups, or varied between age groups as a function of learning block, all *F* ≤ 3.23, *p* ≥ 0.082, η_p_^2^ ≤ 0.092. Thus, older adults' representations of the spatial layout of the environment seem to be more rigidly tied to the sensory input encountered at the beginning of learning, leading to a temporary uncertainty when viewpoints are suddenly changing.

#### Performance in older adults is partly influenced by their facing direction

Age-related differences in pointing performance depending on the nature of the trials during navigational retrieval (i.e., respective intersection-direction-target landmark combination) were further analyzed by means of an ANOVA on the absolute pointing errors with intersection (I1–I4), direction (D1–D4), and target landmark (town hall, church) as repeated measures variables and age group (younger adults, older adults) as between-subjects variable. A significant interaction between the four factors suggested that the performance of the age groups was modulated by the respective intersection-direction-target landmark combination encountered in the VE during retrieval, *F*_(9,288)_ = 2.05, *p* = 0.034, η_p_^2^ = 0.060. Therefore, follow-up ANOVAs were conducted within the two age groups separately. In younger adults, a significant main effect of intersection, *F*_(3,48)_ = 6.18, *p* = 0.005, η_p_^2^ = 0.279 (Greenhouse–Geisser corrected), showed that performance was worse when they were located at I4 (M = 48.6 ± 21.9°) as compared with I1 (M = 25.0 ± 19.7°) or I2 (M = 30.6 ± 25.7°), all *p* ≤ 0.010 (Bonferroni-corrected). This was modulated by a significant interaction between intersection and target landmark, *F*_(3,48)_ = 11.4, *p* < 0.001, η_p_^2^ = 0.416. Pointing errors were smaller in this age group when they pointed toward the town hall (M = 13.5 ± 14.5°) as compared with the church (M = 36.6 ± 29.3°) at I1, which was the intersection adjacent to the town hall, and vice versa at I3, which was the intersection adjacent to the church (town hall: M = 45.5 ± 35.5°; church: M = 25.8 ± 26.9°), all *t* ≥ 3.33, *p* ≤ 0.004, *d* ≥ 0.807. The directions from which the intersections were approached did not have an influence on performance in this age group, all *F* ≤ 1.80, *p* ≥ 0.133, η_p_^2^ ≤ 0.101. In older adults, there was also an interaction between intersection and target landmark, *F*_(3,48)_ = 3.38, *p* = 0.026, η_p_^2^ = 0.174. When located at I3, pointing errors were smaller when the target landmark was the adjacent church (M = 73.2 ± 27.4°) as compared with the town hall (M = 94.2 ± 19.9°), *t*_(16)_ = 2.73, *p* = 0.015, *d* = 0.662. The corresponding comparison for I1 did not reach significance, *t*_(16)_ = 1.12, *p* = 0.281, *d* = 0.271. In addition, there was a significant interaction between direction and target landmark, *F*_(3,48)_ = 3.75, *p* = 0.039, η_p_^2^ = 0.190 (Greenhouse–Geisser corrected). *Post hoc t* tests indicated that pointing toward the town hall (M = 80.3 ± 18.1°) tended to be easier as compared with pointing toward the church (M = 94.5 ± 27.1°) for the older adults when they approached the intersections from D4 (i.e., facing east), *t*_(16)_ = 1.96, *p* = 0.068, *d* = 0.475. In contrast, pointing toward the church (M = 69.9 ± 19.6°) tended to be easier than pointing toward the town hall (M = 87.0 ± 23.3°) when they approached the intersections from D2 (i.e., facing west), *t*_(16)_ = 1.99, *p* = 0.064, *d* = 0.483. This was modulated by an interaction between intersection, direction, and target landmark, *F*_(9,144)_ = 2.25, *p* = 0.022, η_p_^2^ = 0.123. Separate follow-up ANOVAs for each intersection with direction (D1–D4) and target landmark (town hall, church) as repeated measures variables revealed for I2 a main effect of direction, *F*_(3,48)_ = 5.25, *p* = 0.003, η_p_^2^ = 0.247, indicating that pointing generally seemed to be easier from D2 (i.e., facing west; M = 68.8 ± 32.7°) as compared with D1 (M = 94.0 ± 24.5°) or D4 (M = 95.8 ± 37.1°), that is, when they were facing toward the dead-ends at this intersection, all *p* ≤ 0.054 (Bonferroni-corrected). At I3, a main effect of target landmark indicated that pointing toward the adjacent church (M = 73.2 ± 27.4°) was easier for the older adults than pointing toward the town hall (M = 94.2 ± 20.0°), *F*_(3,48)_ = 7.47, *p* = 0.015, η_p_^2^ = 0.318. This was modulated by an interaction between direction and target landmark, *F*_(3,48)_ = 4.44, *p* = 0.008, η_p_^2^ = 0.217. Pointing toward the church was easier when coming from D1 (i.e., facing south; town hall: M = 107.7 ± 31.5°; church: M = 65.6 ± 36.0°) or D2 (i.e., facing west; town hall: M = 98.0 ± 39.4°; church: M = 52.6 ± 40.8°), all *t* ≥ 3.19, *p* ≤ 0.006, *d* ≥ 0.773. Finally, at I4, there was also an interaction between direction and target landmark, *F*_(3,48)_ = 3.74, *p* = 0.017, η_p_^2^ = 0.190. Performance was better when participants pointed toward the church (M = 75.9 ± 30.8°) as compared with the town hall (M = 106.6 ± 31.4°) when approaching the intersection from D2 (i.e., facing west), *t*_(16)_ = 2.59, *p* = 0.020, *d* = 0.629.

To summarize, the results of this analysis again demonstrate better navigational encoding in younger adults and a higher reliance on the specific sensory input in older adults. The directions from which the older adults were approaching the intersections partly seemed to have an impact on their performance, although variability in performance was generally quite high.

#### Individual learning state and response time increase after direction change predict age group

We next used a logistic regression model to check whether age-group can be determined based on two features that characterized age-related performance differences in our task. The mean amount of learning across the whole experiment (i.e., difference between individual learning state estimates from consecutive learning blocks) and the change in response times from the fourth to the fifth learning block served as input features. The model performed very well to estimate the probability of being classified as a younger adult with an average AUC of 0.99 ± 0.02%. Thus, those participants with a higher probability of belonging to the younger age group show better performance on the task while a higher probability of being in the older age group relates to poorer navigational performance, i.e., a lower mean amount of learning across blocks and a higher increase in response times when previously learned locations are encountered from novel viewpoints (Fig. 3*D*).

### fMRI experiment

After preprocessing of the fMRI data using fmriprep (Esteban et al., 2019) and SPM12, we performed a univariate regression analysis to identify age-related differences in neural activity in the RSC/POS and the hippocampus during different phases of the experiment. We further examined the effects of learning at the within-subject and between-subject level. Finally, we examined age-related and learning-related differences in effective connectivity within and between the two regions.

#### Learning ability varies within the older age group

**Figure 4.**
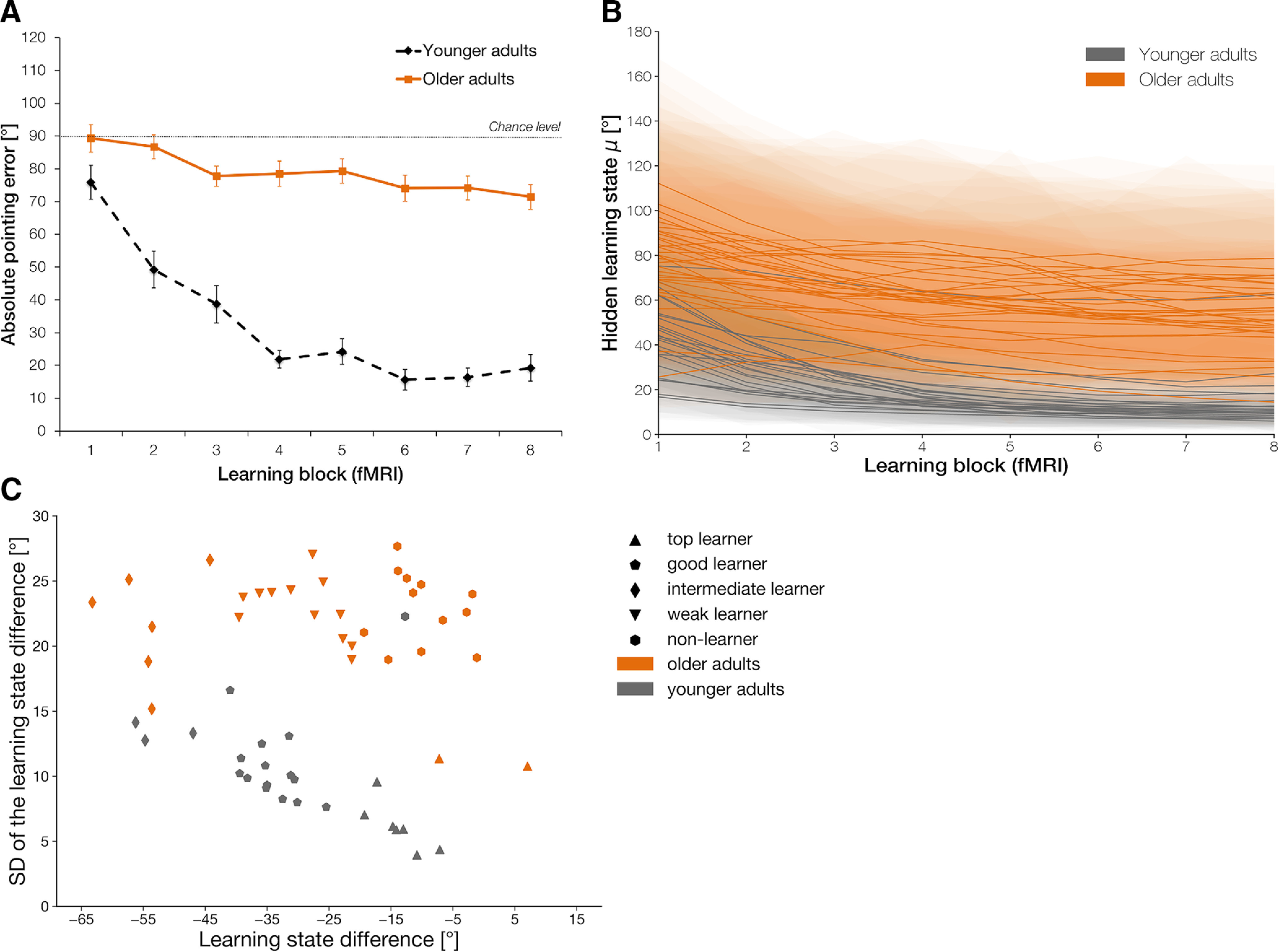
Performance data in the fMRI experiment. ***A***, Average absolute pointing errors across the eight learning blocks in older (solid line) and younger adults (dashed line). Error bars denote SEM. See also Extended Data Figure 3-1*C*,*D* for average pointing errors per learning block for each participant in each age group. ***B***, Mean estimated performance improvement (hidden learning state) of each participant in the older (orange) and younger (gray) age group, including the SD of the posterior distributions (shaded area) across learning blocks. ***C***, Learning subgroups as identified by a K-means clustering algorithm based on the individuals' overall amount of learning and its SD, as determined by the difference of the latent state distributions of the last and first learning block. See Extended Data Figure 4-1 for difference distributions, learning state estimates, and performance data per learning block for representative individuals from each learning subgroup and Extended Data Figure 4-2 for the results of the same clustering analysis within the sample of the behavioral experiment.

As in the behavioral experiment, older compared with younger adults spent considerably more time in the initial familiarization phase of the experiment outside of the scanner (M_old_ = 466 ± 133 s; M_young_ = 258 ± 57.5 s; *t*_(44.3)_ = −7.91, *p* < 0.001, *d* = 2.03. Moreover, we found significant main effects for learning block, *F*_(7,385)_ = 32.3, *p* < 0.001, η_p_^2^ = 0.370, and age group, *F*_(1,55)_ = 167, *p* < 0.001, η_p_^2^ = 0.752, together with a significant interaction between the two factors for the average absolute pointing errors, *F*_(7,385)_ = 11.0, *p* < 0.001, η_p_^2^ = 0.166. This indicates that younger compared with older adults again showed better performance on the task and stronger improvement across learning blocks (Fig. 4*A*). Older adults, however, did show learning at the group level as confirmed by a separate ANOVA within this age group, *F*_(7,217)_ = 3.58, *p* = 0.001, η_p_^2^ = 0.103. Accuracy for the control trials was at ceiling across the whole sample (mean proportion of correct responses = 0.97 ± 0.05). In contrast to the behavioral experiment, the change in directions from the first to the second half of the experiment was omitted here because of the reduced number of trials per learning block. Thus, we did not expect changes in response times from the first to the second half of the experiment.

10.1523/JNEUROSCI.0528-20.2021.f4-1 Extended Data Figure 4-1 Definition of learning subgroups. Hidden learning states (including SD) and trial-wise performance data per learning block (left) and the latent state distributions of the last and first learning block plotted together with the difference distribution (right) from representative individuals from each learning subgroup in the fMRI experiment. Download Figure 4-1, TIF file.

10.1523/JNEUROSCI.0528-20.2021.f4-2 Extended Data Figure 4-2 Learning subgroups in the behavioral experiment as identified by a K-means clustering algorithm based on the individuals' overall amount of learning and its SD, as determined by the difference of the latent state distributions of the last and first learning block. Results are shown for (***A***) five and (***B***) six learning clusters that yielded similar silhouette scores (respective mean silhouette scores per tested cluster number: 3: 0.232, 4: 0.293, 5: 0.400, 6: 0.404, 7: 0.370). Download Figure 4-2, TIF file.

10.1523/JNEUROSCI.0528-20.2021.f4-3 Extended Data Figure 4-3 Key demographics of the learning subgroups within each age group in the fMRI experiment. Download Figure 4-3, DOCX file.

Individual learning state estimates as obtained from the state-space model again showed that participants varied substantially in their ability to learn the spatial layout of the VE (Fig. 4*B*; see also Extended Data Fig. 3-1*C*,*D* for average pointing errors per learning block for each participant). To determine how neural activation patterns were modulated by the individuals' overall amount of learning across the experiment, we used a K-means clustering algorithm to identify learning subgroups based on the difference between the latent state distributions of the last and first learning block. The estimated optimal number of clusters in our sample turned out to be five (Fig. 4*C*). A group of top learners (*n* = 9), consisting of seven younger adults and two older adults, already learned the layout of the VE after the familiarization phase resulting in a small difference in learning between the first and the last learning block. The second cluster exclusively consisted of younger adults, categorized as good learners (*n* = 14). They typically reached ceiling performance during the first half of the experiment with a low variance in their difference distribution. A group of intermediate learners (*n* = 9), consisting of a three younger and six older adults, were still improving in the second half of the experiment and consequently exhibited the largest difference in their hidden learning state from the beginning to the end of the experiment and a relatively high variance. Individuals belonging to the fourth cluster were categorized as weak learners (*n* = 12) who showed only a small improvement across the whole experiment and a high variability. This cluster consisted of older adults only. Finally, 12 older adults and one younger adult did not show considerable improvement across the learning blocks and were consequently categorized as non-learners (*n* = 13) in the context of our experiment. Difference distributions for representative individuals from each learning subgroup, together with learning estimates and behavioral data per learning block, can be found in Extended Data Fig. 4-1. Although these results are specific to our sample, the same clustering analysis within the behavioral experiment yielded comparable results in terms of the number of learning subgroups, underscoring the validity of its results in the context of our task (see Extended Data Fig. 4-2).

These between-subject differences in learning demonstrate that our task was neither too easy nor too difficult for one of the age groups per se. In addition, the learning subgroups within each age group were comparable with respect to the factors age, sex or signs of major cognitive impairment (Extended Data Fig. 4-3).

#### Age-related hyperactivation in the hippocampus and RSC/POS during navigational retrieval

First, to identify overall age-related differences in activation patterns within the RSC/POS and the hippocampus, regardless of learning, we contrasted navigational retrieval trials to control trials. Activity in medial parts of the RSC/POS was increased in older compared with younger adults for this comparison. This age-related activity increase was also observed in the left anterior hippocampus (Table 1, increased activity in older compared with younger adults during navigation vs control). An age-related activity reduction was found in a small cluster in the superior right POS and also in a more lateral cluster in the right POS (Table 1, reduced activity in older compared with younger adults during navigation vs control). Second, we tested for interactions between age group and activation differences during navigational retrieval versus encoding to check whether these two phases of the experiment were differently affected by age. In one cluster of the right POS as well as two clusters in the right and left anterior hippocampus, activity was increased in older adults compared with younger adults during navigational retrieval versus encoding (Table 1, increased activity in older compared with younger adults during retrieval vs encoding). There were no clusters within our ROIs where activity was reduced in older adults. Activations outside of our ROIs for these two comparisons and the corresponding results for the whole sample can be found in Extended Data Table 1-1.

**Table 1.** Spatial coordinates of the local maxima in the hippocampus and RSC/POS ROIs in the fMRI analyses on age-related differences in neural activation patterns (*p* < 0.05, FWE-corrected)

Brain region	Cluster size	MNI coordinate			*Z*-score
		*x*	*y*	*z*	
Increased activity in older compared with younger adults during navigation vs control					
L POS	83	−3	−60	34	4.56
L RSC		−6	−57	21	4.32
L Hippocampus	72	−21	−18	−12	4.76
		−30	−15	−22	4.21
Reduced activity in older compared with younger adults during navigation vs control					
R POS	22	12	−69	54	4.00
		18	−69	57	3.94
		15	−75	51	3.25
R POS	55	27	−60	24	3.96
Increased activity in older compared with younger adults during retrieval vs encoding					
R POS	27	12	−51	34	4.03
L Hippocampus	33	−30	−15	−15	4.08
R Hippocampus	20	24	−12	−12	3.97
Age-group differences in learning-related activity decreases					
R Hippocampus	20	24	−18	−15	4.55
Age-group differences in learning-related activity increases					
L POS	462	−9	−66	24	5.93
		−24	−72	47	5.37
L RSC		−18	−57	1	4.14
		−6	−63	11	3.98
R POS	148	24	−69	47	4.95
		21	−72	54	4.73
R RSC	205	9	−57	4	4.82
		9	−63	21	4.59

See Extended Data Table 1-1 for significantly activated clusters elsewhere in the brain.

10.1523/JNEUROSCI.0528-20.2021.tab1-1 Extended Data Table 1-1 Spatial coordinates of the local maxima in the whole-brain fMRI analyses on age-related differences in neural activation patterns (*p* < 0.05, whole-brain FWE-corrected). Download Table 1-1, DOCX file.

#### Learning-related activity changes in anterior hippocampus and RSC/POS are less pronounced in older adults

**Figure 5.**
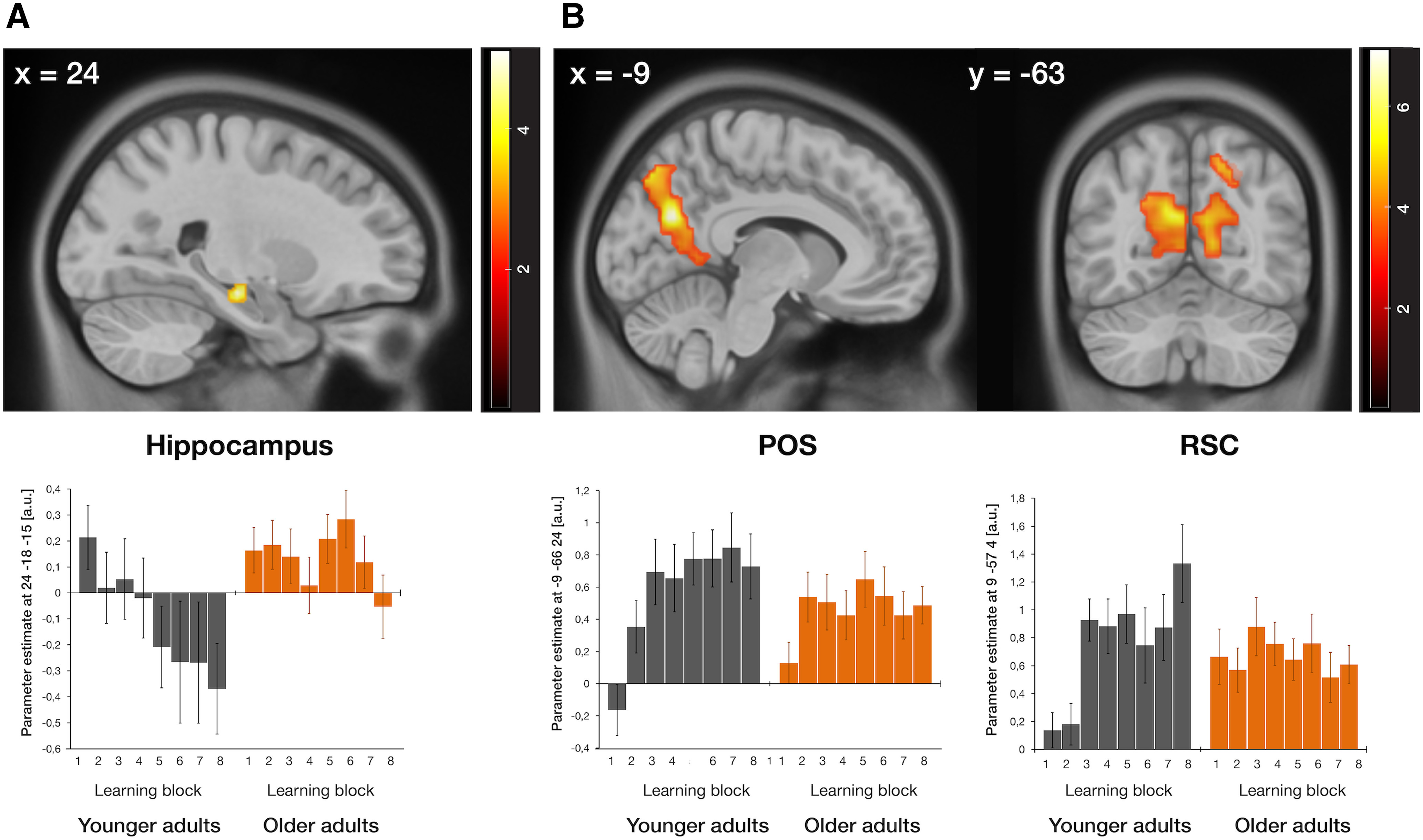
Interaction effects between age group and the amount of learning per block during navigational retrieval. Age-related differences in (***A***) hippocampal activity decreases and (***B***) RSC/POS activity increases across the experiment. Activations are displayed on the 2009 nonlinear asymmetric MNI template that was used for normalization (*p* < 0.05, FWE-corrected for the respective ROI). Plots depict average parameter estimates of the respective peak voxels per learning block in selected clusters for each age group. Error bars indicate the across-subject standard error of the mean. See Table 1 for the spatial coordinates of the local maxima in the hippocampus and RSC/POS ROIs and Extended Data Table 1-1 for significantly activated clusters elsewhere in the brain.

By using the amount of learning per block as contrast weights in our GLM, we assessed learning-related age-group differences in the time course of hippocampal and RSC/POS involvement during navigational retrieval. First, we found that activity in the anterior portion of the right hippocampus decreased in younger but less so in older adults as a function of learning (Table 1, age-group differences in learning-related activity decreases; Fig. 5*A*). This suggests that hippocampal activity reflected the amount of spatial knowledge that was acquired after each encoding tour in the younger age group. In older adults, in contrast, hippocampal activation did not change systematically across learning blocks. Second, we also found several clusters within the RSC/POS ROI where activity increased over the course of the experiment more in younger than in older adults (Table 1, age-group differences in learning-related activity increases; Fig. 5*B*). This concerned the whole extent of the left POS from its superior parts to the left RSC, a cluster in the right RSC/POS, and a more lateral cluster in the right POS. Activity in these clusters paralleled changes in performance across learning blocks in the younger age group. Older adults' individual learning curves, in contrast, were again decoupled from activity changes in these regions. Activations outside of our ROIs for these comparisons and the corresponding results for the whole sample can be found in Extended Data Table 1-1.

10.1523/JNEUROSCI.0528-20.2021.f5-1 Extended Data Figure 5-1 Spatial coordinates of the local maxima in the whole-brain fMRI analyses on age-related differences in neural activation patterns (*p* < 0.05, whole-brain FWE-corrected). Download Figure 5-1, DOCX file.

It is possible that the decreasing BOLD responses in the hippocampus and the increasing responses in RSC/POS, which we observed in younger adults, could have been driven by younger adults becoming quicker with self-localization, allowing them to compute the direction toward the target landmark at progressively earlier time points. Under this scenario, one would predict a temporal shift of the BOLD response, in particular for the RSC (assuming a role of the RSC/POS in deriving directional relationships between one's position and landmarks). To directly test this hypothesis, we performed a control analysis using a FIR model of the hemodynamic response but did not find any indications that the time bin of the peak of the HRF changed between the first and the second half of the experiment, neither in the hippocampus nor in the RSC/POS ROI. In the hippocampus, the median time bin was 3.75 (IQR = 1.25) in the first and 4.25 (IQR = 1.50) in the second half of the experiment in younger adults (out of eight time bins that were modeled with a 2-s duration each). In older adults, the median in the first half was 4.50 (IQR = 1.44) and 4.50 (IQR = 0.75) in the second half. A two-sample Wilcoxon test confirmed that these differences were not significant in any of the experiment halves (all *p* ≥ 0.106, Bonferroni corrected, all effect sizes *r* ≤ 0.262). Similar results were obtained in the RSC/POS ROI with a median of 4.75 (IQR = 1.00) in the first and 4.50 (IQR = 1.00) in the second half within the younger age group and 4.50 (IQR = 1.19) in the first and 4.75 (IQR = 1.25) in the second half within the older age group (all *p* ≥ 1.00, Bonferroni corrected, all effect sizes *r* ≤ 0.049). This suggests that the differential hippocampal and RSC/POS dynamics in the two age groups are unlikely to be driven by changes in the onset/duration of the spatial computations conducted in the two regions.

#### Learning-related activity changes across blocks are modulated by interindividual differences in learning within older adults

**Figure 6.**
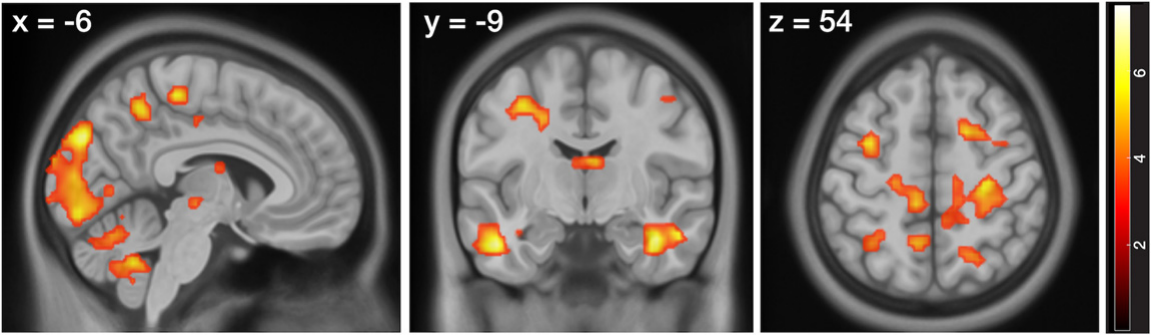
Differential activity changes in relation to the amount of learning per block between learning subgroups in the older age group. Activations are displayed on the 2009 nonlinear asymmetric MNI template that was used for normalization (*p* < 0.05, FWE-corrected). See Extended Data Figure 6-1 for the spatial coordinates of the local maxima.

Behavioral performance of older adults varied substantially, with some of them showing hidden learning states similar to younger adults while others showed very little performance improvements. Therefore, we next included the individual's learning subgroup as covariate in the second-level analysis to examine in which regions learning-related activity changes across blocks differed as a function of the overall learning ability of the individual. In younger adults, no activations emerged within our ROIs or elsewhere in the brain. In older adults, however, we found that activity changes in several regions across the entire brain, including visual cortices, the cerebellum, temporal and frontal cortices, as well as the parahippocampal cortex (PHC) extending to the anterior hippocampus, were more strongly related to the individual learning curves in better performing groups (i.e., decreased across learning blocks; Fig. 6; Extended Data Fig. 6-1). The learning curves of those older adults who were less able to learn the layout of the environment, in contrast, were decoupled from activity changes in these regions. No activations survived our correction for multiple comparisons within our ROIs or across the whole-brain when testing for the interactions between age group and learning subgroup.

10.1523/JNEUROSCI.0528-20.2021.f6-1 Extended Data Figure 6-1 Spatial coordinates of the local maxima in the fMRI analyses on inter-individual differences in neural activation patterns across learning blocks within older adults (*p* < 0.05, FWE-corrected). Download Figure 6-1, DOCX file.

#### Age-related reduction in the inhibitory self-connection of the anterior hippocampus

To check whether age-related problems in spatial learning are related to changes in the intrinsic excitability of the anterior hippocampus and the RSC/POS or in the coupling between the two regions, we used DCM PEB (Friston et al., 2016). DCM has been successfully used to determine effective connectivity changes in the hippocampus and related regions during memory processing (Gluth et al., 2015). Moreover, DCM PEB offers several advantages over classical DCM variants in terms of model selection and second-level group comparisons. First, instead of specifying several models at the first level and comparing their evidence, a full model is estimated for each participant incorporating all parameters of interest, and BMR is performed to obtain posterior estimates of nested models in which parameters that do not contribute to the model evidence are pruned. Second, first-level DCMs are equipped with empirical priors that shrink parameter estimates toward a group mean. In this way, each subject's contribution to the group PEB result is weighted by their precision. Third, applying classical inference methods to examine whether certain parameters differ between groups after model inversion ignores within-subject uncertainty (i.e., variance of the posterior distributions). This is circumvented in PEB by using the full posterior density over the parameters from each participant's DCM to draw inferences about group level effects.

**Figure 7.**
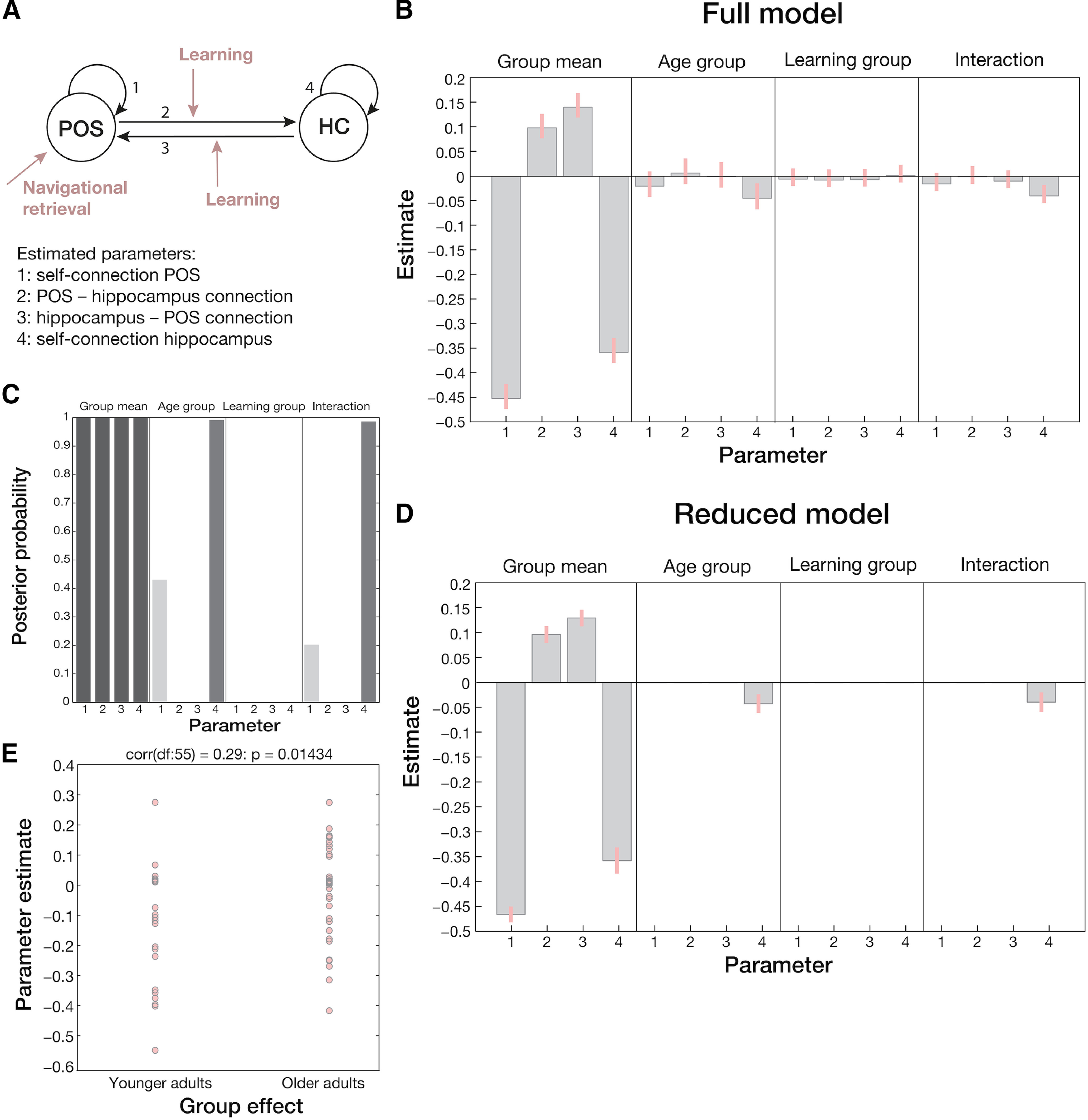
Results of the DCM PEB analysis. ***A***, First-level DCM specification to determine average connectivity within and between anterior hippocampus and POS. Navigational retrieval phases were modeled as driving input entering the cortical network via the POS, and the amount of learning per block was included as modulatory input on the bidirectional connections between the regions. Estimated parameters (1: self-connection POS, 2: POS–hippocampus connection, 3: hippocampus–POS connection, 4: self-connection hippocampus) before (***B***) and after (***D***) BMR for each covariate (age group, learning group, interaction between age group, and learning group) in the second-level PEB model. Gray bars represent parameter means and pink lines their 95% confidence intervals. The parameters for self-connections (parameters 1 and 4) are expressed as log scaling parameters that can be converted to Hz using x_Hz = −0.5 * exp(x) whereby × is the log scaling parameter and −0.5 Hz the prior. ***C***, Posterior probabilities per parameter for each second-level covariate after BMR. ***E***, Predicted age group of each participant as derived from a LOO cross-validation scheme based on the estimated self-connection strength in the anterior hippocampus.

For each participant, we first specified and estimated a DCM between the anterior hippocampus and the POS using peak coordinates from the corresponding univariate analysis. Navigational retrieval phases were modeled as driving input into the network via the POS. The amount of learning per block was modeled as modulatory input on the bidirectional connections between the two regions (Fig. 7*A*). In the second-level PEB model, we included age group, learning subgroup, and their interaction as covariates to determine their relative influence on the connection strengths. Figure 7, left panels, shows the group mean of the average connection strength before (Fig. 7*B*) and after BMR (Fig. 7*D*), indicating that all four parameters were necessary to explain our data.

With respect to age group differences in connectivity, only one parameter survived BMR (Fig. 7*B*,*D*, second panels). Specifically, older compared with younger adults had a reduced inhibitory self-connection strength in the anterior hippocampus, i.e., a relative disinhibition in this region. Note that for self-connections in the DCM framework, parameters are expressed as log scaling parameters and that the regressor representing age group was coded in a way that the resulting parameter is the amount that needs to be added to the group mean to obtain the older adults' connection strength (the group mean is obtained by calculating −0.5 Hz * exp(−0.33698) = −0.357 Hz and for older adults −0.5 Hz * exp(−0.33698 + −0.039719) = −0.3431 Hz). Thus, our model provides evidence that the aging hippocampus seems to be more readily excited by afferent activity from other regions during spatial learning. The interaction between age group and learning subgroup in this model parameter also survived BMR (Fig. 7*B*,*D*, right panels), indicating that the hippocampal self-connection strength was more strongly modulated by the overall learning ability of the individual in the older age group. Inspection of Figure 7*D*, forth panel, indicates that the age-related disinhibition in this region was attenuated in better performing individuals (see also Fig. 7*C* for posterior probabilities of each parameter). We did not find any modulatory effects of the (within-subject) amount of learning per block.

We further performed a LOO cross-validation using the model parameter denoting the self-connection strength in the anterior hippocampus to test whether this effect would be large enough to predict the participants' age group. In this analysis, all but one subject were used to estimate the model parameter, which was then used to evaluate the posterior belief of the model parameter in a left-out (test) subject. The predicted and actual between-subject effect for each test subject was then compared with derive an independent out-of-sample correlation, which was 0.29 in the current sample (*p* = 0.01434; Fig. 7*E*). Thus, the estimated intrinsic connection strength in the anterior hippocampus during spatial learning was large enough to predict the age group of a new subject above chance level.

### Summary of the key findings

**Figure 8.**
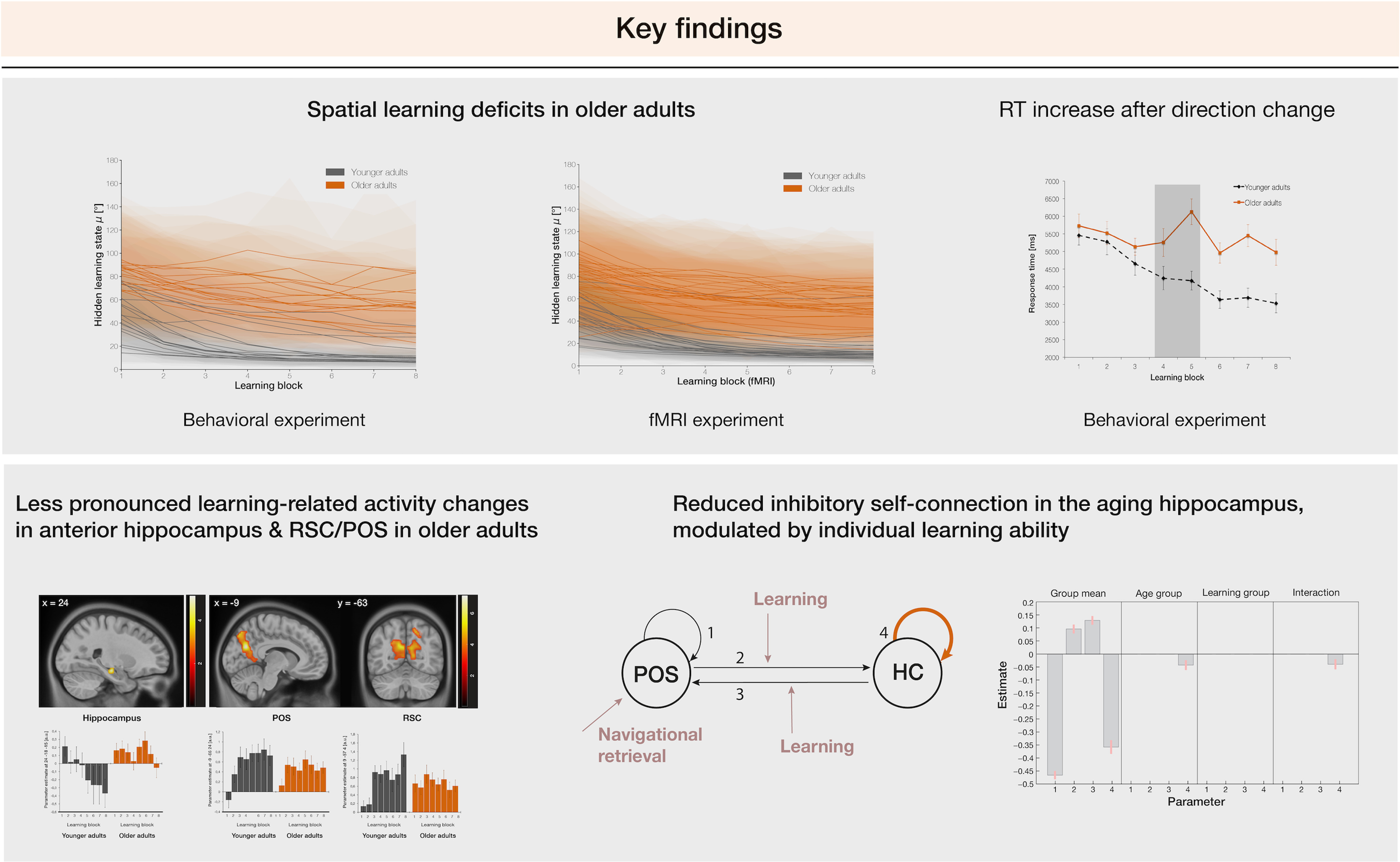
Key findings of the two experiments.

At the behavioral level, we found in two separate experiments that performance improvements were considerably reduced in healthy older compared with younger adults, when they were asked to retrieve the spatial layout of an initially unfamiliar environment. Older adults further showed a higher uncertainty when familiar locations were experienced from novel viewpoints during learning, as evidenced by a temporary increase in response times. At the neural level, activity in the anterior hippocampus and RSC/POS changed dynamically as a function of learning in younger adults, whereas this was not the case in older adults. Importantly, a DCM PEB analysis revealed that the inhibitory self-connection of the anterior hippocampus was reduced in older adults and was modulated by the overall learning ability of the individual as evidenced by an interaction between age group and learning subgroup (see Fig. 8 for a graphical summary of the results).

## Discussion

In two experiments, we show that healthy older adults, on average, have substantial problems in learning to orient themselves in a novel, city-like VE, in line with previous findings (Iaria et al., 2009; Yamamoto and DeGirolamo, 2012). At neural levels, we could replicate earlier findings showing that activity in RSC/POS increased while activity in the anterior hippocampus decreased as a function of learning in younger adults (Wolbers and Büchel, 2005; Auger et al., 2015; Brodt et al., 2016), which shows that our task is suitable to measure spatial learning, while using a complex photorealistic VE. In older adults, activity in these two regions was decoupled from the amount of learning and did not change systematically across repeated episodes in the environment. Importantly, we provide the first evidence that an increased excitability of the anterior hippocampus might constitute a potential neural mechanism for cognitive mapping deficits in older adults.

In the behavioral experiment, we additionally found that older adults had problems when locations are encountered from novel directions during learning. This might be related to age-related deficits in distinguishing novel from familiar input (Yassa et al., 2011; Vieweg et al., 2015) and to impairments in allocentric processing, because Wiener et al. (2013) observed age-related performance declines when locations were approached from novel directions during route learning. Given that viewpoint transformations in spatial memory involve hippocampal computations (King et al., 2002), the behavioral results already point to impaired information processing within the aging hippocampus that affects navigational learning. This extends findings showing that a reduced sensitivity to changes in the environment might be linked to age-related impairments in object-location binding and spatial perspective taking (Muffato et al., 2019; Segen et al., 2021).

In the fMRI experiment, performance relied on the knowledge about the relation between the participant's own position and the position of the target landmarks, while the change in viewpoints was omitted. What neural mechanisms can account for the cognitive mapping deficits in older adults? The learning-related activity decrease in the anterior hippocampus of younger adults was absent in older adults, leading to an overall hippocampal hyperactivity. Similar effects have been observed in studies investigating age-related deficits in pattern separation (Yassa et al., 2011; Reagh et al., 2018), as well as in rodent and non-human primate studies on age-related changes in spatial navigation (Wilson et al., 2005; Thomé et al., 2016). By examining effective connectivity, we were able to show, for the first time, that an age-related reduction in the inhibitory self-connection strength of the anterior hippocampus might constitute the underlying neural mechanism for the elevated signal in this region. Within the context of DCM, self-connection parameters capture, at a macroscopic level, condition specific changes in the excitatory-inhibitory balance (Friston et al., 2019). Because effective connectivity as inferred using DCM for fMRI is typically polysynaptic, we cannot determine which class of cells or synapses underlie these effects. In memory-impaired monkeys, increased firing rates in CA3 place cells have been linked to a reduced number of GABAergic inhibitory interneurons (Thomé et al., 2016). Whether this is similarly the case in humans and how this is related to AD pathogenesis are important questions for future research (Bi et al., 2020).

The age effect on the hippocampal self-connection strength was modulated by the learning ability of the individual, suggesting that an increased hippocampal excitability might impair the formation of spatial knowledge. Specifically, aberrant activity in the hippocampus could have affected the spatial resolution of the emerging cognitive maps in older adults, in line with findings showing that (1) hippocampal lesion patients and healthy older adults are impaired in forming high-resolution spatial representations when navigating novel environments (Kolarik et al., 2016, 2018; Nilakantan et al., 2018); and (2) that reducing hippocampal hyperactivity with an anti-epileptic drug that targets excitatory neurotransmission improves memory performance in amnestic patients (Bakker et al., 2015; see also Koh et al., 2013; Robitsek et al., 2015 for related findings in rodents). Critically, in the context of our task, imprecise cognitive maps will not only affect self-localization but also the ability to compute allocentric vectors to the target landmarks. The latter process has also been linked to computations in subregions of the MTL (Chadwick et al., 2015; Shine et al., 2019; see also Wang et al., 2018; Høydal et al., 2019).

In addition to hippocampal hyperactivation, older adults also exhibited a lack of learning-related dynamics in RSC/POS. Medial parietal cortex undergoes significant changes during aging, including increased atrophy and enhanced tau deposition (Jockwitz et al., 2017; Harrison et al., 2019). Moreover, the increased excitability of the aging hippocampus may impact on information processing in RSC/POS, given the close reciprocal interactions between both regions. For example, Mao et al. (2018) found that bilateral hippocampal lesions suppress the gradual emergence of a spatial code in the RSC. In the present study, given that RSC/POS is assumed to support the anchoring of cognitive maps to external landmarks (Epstein et al., 2017), a deficient anchoring may compromise older adults' ability to precisely recover their facing direction and to orient their cognitive maps when approaching the intersections. Together with the imprecision in the cognitive maps, both deficits are likely to contribute to the compromised pointing performance in older adults.

Moreover, this anchoring process should occur in parallel to self-localization in our task, because as soon as an intersection was visible during navigational retrieval, participants could use the local buildings and/or the geometric layout to recover both their position and their facing direction. This could explain why, particularly in younger adults, the latency of the BOLD response in RSC/POS did not change over the course of the experiment, because the process of reorientation could start immediately at the beginning of a trial.

Our univariate results differ from Moffat et al. (2006) who measured brain activity during encoding of a virtual maze and reported an age-related hypoactivation in the RSC and the hippocampus. This discrepancy might be related to the time point when activity was measured, because if younger adults were still acquiring knowledge about the VE, our findings would also predict stronger hippocampal effects compared with older adults. More generally, this discrepancy highlights the need to track the learning status of an individual when interpreting differences in (hippocampal) BOLD responses between groups. In addition, it is important to note that we focused on hemodynamic changes during retrieval in our study. Thus, overall task demands could be another factor that might have contributed to our findings, because we also observed an age-related activity increase in RSC/POS and hippocampus when contrasting retrieval to encoding.

Performance in our task was highly variable. While some older adults learned the layout of the environment as quickly as younger adults, others showed continuous learning, learned very slowly, or were not able to retrieve relevant information to perform the task. During MRI scanning, the amount of exposure in the VE was kept constant for all participants. This allowed us to replicate earlier findings in younger adults and to use this as a baseline against which we could compare the results of the older adults. Therefore, we cannot determine whether low-performing older adults would just need more time for learning. However, it seems unlikely that all of them would have reached the same performance level as younger adults if provided with more time in the VE, because older adults already spent considerably more time in the initial familiarization phase of the experiments. Using machine learning methods on MRI data of hundreds of older adults, Eavani et al. (2018) described multiple phenotypes of brain agers that are characterized by specific functional and structural changes. The authors described one phenotype that displays atrophy in the hippocampus, decreased coherence in posterior medial parietal cortex, and an increased connectivity in the MTL. Thus, older adults who show an increased excitability of the anterior hippocampus might be particularly impaired in memorizing novel spatial environments.

Finally, by forming subgroups of learners based on their estimated learning states and by including this information in the fMRI analysis, we found that activity changes in several brain regions were decoupled from the individual learning curves in those older adults who had more problems to learn. Although these results should be interpreted with caution given the small sample sizes of our groups, they provide further indications that hyperactivity in the aging brain does not seem to support task performance (Morcom and Henson, 2018). We did not find any indications that the learning differences within older adults were related to their age, sex, or their cognitive screening scores. Thus, future studies should apply additional measures, for example, preclinical markers for AD, to further characterize age-related deficits in spatial learning and, specifically, why these abilities are preserved in some older adults.

Taken together, increased excitability of the anterior hippocampus, together with aberrant RSC/POS functioning, provides a novel explanation why older adults experience problems with forming accurate spatial representations of a novel environment. In addition, our findings add to a growing body of evidence associating hyperactivity in the hippocampus to memory impairments in aging.

## References

[B1] Auger SD, Zeidman P, Maguire EA (2015) A central role for the retrosplenial cortex in de novo environmental learning. Elife 4:e09031. 10.7554/eLife.09031 PMC4559753 26284602

[B2] Bakker A, Albert MS, Krauss G, Speck CL, Gallagher M (2015) Response of the medial temporal lobe network in amnestic mild cognitive impairment to therapeutic intervention assessed by fMRI and memory task performance. Neuroimage Clin 7:688–698. 10.1016/j.nicl.2015.02.009 25844322 PMC4377841

[B3] Barnes CA, Suster MS, Shen J, McNaughton BL (1997) Multistability of cognitive maps in the hippocampus of old rats. Nature 388:272–275. 10.1038/40859 9230435

[B4] Behzadi Y, Restom K, Liau J, Liu TT (2007) A component based noise correction method (CompCor) for BOLD and perfusion based fMRI. Neuroimage 37:90–101. 10.1016/j.neuroimage.2007.04.042 17560126 PMC2214855

[B5] Berens P (2009) CircStat: a MATLAB toolbox for circular statistics. J Stat Softw 31:1–21.

[B6] Bi D, Wen L, Wu Z, Shen Y (2020) GABAergic dysfunction in excitatory and inhibitory (E/I) imbalance drives the pathogenesis of Alzheimer's disease. Alzheimers Dement 16:1312–1329. 10.1002/alz.12088 32543726

[B7] Braak H, Del Tredici K (2015) The preclinical phase of the pathological process underlying sporadic Alzheimer's disease. Brain 138:2814–2833. 10.1093/brain/awv236 26283673

[B8] Brett M, Anton JL, Valabregue R, Poline JB (2002) Region of interest analysis using an SPM toolbox. Neuroimage 13:210–217.

[B9] Brodt S, Pöhlchen D, Flanagin VL, Glasauer S, Gais S, Schönauer M (2016) Rapid and independent memory formation in the parietal cortex. Proc Natl Acad Sci USA 113:13251–13256. 10.1073/pnas.1605719113 27803331 PMC5135314

[B10] Bzdok D, Heeger A, Langner R, Laird AR, Fox PT, Palomero-Gallagher N, Vogt BA, Zilles K, Eickhoff SB (2015) Subspecialization in the human posterior medial cortex. Neuroimage 106:55–71. 10.1016/j.neuroimage.2014.11.009 25462801 PMC4780672

[B11] Carpenter B, Gelman A, Hoffman MD, Lee D, Goodrich B, Betancourt M, Brubaker M, Guo J, Li P, Riddell A (2017) Stan: a probabilistic programming language. J Stat Softw 76:1–32. 10.18637/jss.v076.i01 PMC9788645 36568334

[B12] Chadwick MJ, Jolly AEJ, Amos DP, Hassabis D, Spiers HJ (2015) A goal direction signal in the human entorhinal/subicular region. Curr Biol 25:87–92. 10.1016/j.cub.2014.11.001 25532898 PMC4291144

[B13] Commandeur J, Koopman SJ (2007) Introduction to state space time series analysis. Oxford: Oxford University Press.

[B14] Dale AM, Fischl B, Sereno MI (1999) Cortical surface-based analysis: I. Segmentation and surface reconstruction. Neuroimage 9:179–194. 10.1006/nimg.1998.0395 9931268

[B15] Daunizeau J, Stephan KE, Friston KJ (2012) Stochastic dynamic causal modelling of fMRI data: should we care about neural noise? Neuroimage 62:464–481. 10.1016/j.neuroimage.2012.04.061 22579726 PMC3778887

[B16] Destrieux C, Fischl B, Dale AM, Halgren E (2010) Automatic parcellation of human cortical gyri and sulci using standard anatomical nomenclature. Neuroimage 53:1–15. 10.1016/j.neuroimage.2010.06.010 20547229 PMC2937159

[B17] Diersch N, Wolbers T (2019) The potential of virtual reality for spatial navigation research across the adult lifespan. J Exp Biol 222:jeb187252. 10.1242/jeb.187252 30728232

[B18] Eavani H, Habes M, Satterthwaite TD, An Y, Hsieh MK, Honnorat N, Erus G, Doshi J, Ferrucci L, Beason-Held LL, Resnick SM, Davatzikos C (2018) Heterogeneity of structural and functional imaging patterns of advanced brain aging revealed via machine learning methods. Neurobiol Aging 71:41–50. 10.1016/j.neurobiolaging.2018.06.013 30077821 PMC6162110

[B19] Epstein RA (2008) Parahippocampal and retrosplenial contributions to human spatial navigation. Trends Cogn Sci 12:388–396. 10.1016/j.tics.2008.07.004 18760955 PMC2858632

[B20] Epstein RA, Patai EZ, Julian JB, Spiers HJ (2017) The cognitive map in humans: spatial navigation and beyond. Nat Neurosci 20:1504–1513. 10.1038/nn.4656 29073650 PMC6028313

[B21] Esteban O, Birman D, Schaer M, Koyejo OO, Poldrack RA, Gorgolewski KJ (2017) MRIQC: advancing the automatic prediction of image quality in MRI from unseen sites. PLoS One 12:e0184661 10.1371/journal.pone.0184661 28945803 PMC5612458

[B22] Esteban O, Markiewicz C, Blair RW, Moodie C, Isik AI, Erramuzpe AA, Kent J, Goncalves M, DuPre E, Snyder M, Oya H, Ghosh SS, Wright JD, Durnez J, Poldrack RA, Gorgolewski KJ (2019) FMRIPrep: a robust preprocessing pipeline for functional MRI. Nat Methods 16:111–116. 10.1038/s41592-018-0235-4 30532080 PMC6319393

[B23] Faul F, Erdfelder E, Lang AG, Buchner A (2007) G*Power 3: a flexible statistical power analysis program for the social, behavioral, and biomedical sciences. Behav Res Methods 39:175–191. 10.3758/bf03193146 17695343

[B24] Fischl B, Salat DH, Busa E, Albert M, Dieterich M, Haselgrove C, van der Kouwe A, Killiany R, Kennedy D, Klaveness S, Montillo A, Makris N, Rosen BR, Dale AM (2002) Whole brain segmentation: automated labeling of neuroanatomical structures in the human brain. Neuron 33:341–355. 10.1016/S0896-6273(02)00569-X 11832223

[B25] Fischl B, Sereno MI, Dale AM (1999) Cortical surface-based analysis: II: inflation, flattening, and a surface-based coordinate system. Neuroimage 9:195–207. 10.1006/nimg.1998.0396 9931269

[B26] Friston KJ, Litvak V, Oswal A, Razi A, Stephan KE, van Wijk BCM, Ziegler G, Zeidman P (2016) Bayesian model reduction and empirical Bayes for group (DCM) studies. Neuroimage 128:413–431. 10.1016/j.neuroimage.2015.11.015 26569570 PMC4767224

[B27] Friston KJ, Preller KH, Mathys C, Cagnan H, Heinzle J, Razi A, Zeidman P (2019) Dynamic causal modelling revisited. Neuroimage 199:730–744. 10.1016/j.neuroimage.2017.02.045 28219774 PMC6693530

[B28] Gelman A, Shirley K (2011) Inference from simulations and monitoring convergence. In: Handbook of Markov Chain Monte Carlo (Brooks S, Gelman A, Jones GL, Meng XL, eds), pp 163–174. Boca Raton: Chapman Hall.

[B29] Gluth S, Sommer T, Rieskamp J, Büchel C (2015) Effective connectivity between hippocampus and ventromedial prefrontal cortex controls preferential choices from memory. Neuron 86:1078–1090. 10.1016/j.neuron.2015.04.023 25996135

[B30] Gorgolewski KJ, Auer T, Calhoun VD, Craddock RC, Das S, Duff EP, Flandin G, Ghosh SS, Glatard T, Halchenko YO, Handwerker DA, Hanke M, Keator D, Li X, Michael Z, Maumet C, Nichols BN, Nichols TE, Pellman J, Poline JB, et al. (2016) The brain imaging data structure, a format for organizing and describing outputs of neuroimaging experiments. Sci Data 3:160044. 10.1038/sdata.2016.44 27326542 PMC4978148

[B31] Grady CL (2012) The cognitive neuroscience of ageing. Nat Rev Neurosci 13:491–505. 10.1038/nrn3256 22714020 PMC3800175

[B32] Grinband J, Steffener J, Razlighi QR, Stern Y (2017) BOLD neurovascular coupling does not change significantly with normal aging. Hum Brain Mapp 38:3538–3551. 28419680 10.1002/hbm.23608 PMC5882590

[B33] Harrison TM, La Joie R, Maass A, Baker SL, Swinnerton K, Fenton L, Mellinger TJ, Edwards L, Pham J, Miller BL, Rabinovici GD, Jagust WJ (2019) Longitudinal tau accumulation and atrophy in aging and Alzheimer disease. Ann Neurol 85:229–240. 10.1002/ana.25406 30597624 PMC6579738

[B34] Høydal ØA, Skytøen ER, Andersson SO, Moser MB, Moser EI (2019) Object-vector coding in the medial entorhinal cortex. Nature 568:400–404. 10.1038/s41586-019-1077-7 30944479

[B35] Iaria G, Palermo L, Committeri G, Barton JJS (2009) Age differences in the formation and use of cognitive maps. Behav Brain Res 196:187–191. 10.1016/j.bbr.2008.08.040 18817815

[B36] Jockwitz C, Caspers S, Lux S, Jütten K, Schleicher A, Eickhoff SB, Amunts K, Zilles K (2017) Age- and function-related regional changes in cortical folding of the default mode network in older adults. Brain Struct Funct 222:83–99. 10.1007/s00429-016-1202-4 26943919

[B37] Jones E, Oliphant T, Peterson P (2001) SciPy: open source scientific tools for Python. Available at http://www.scipy.org.

[B38] King JA, Burgess N, Hartley T, Vargha-Khadem F, O'Keefe J (2002) Human hippocampus and viewpoint dependence in spatial memory. Hippocampus 12:811–820. 10.1002/hipo.10070 12542232

[B39] Kobayashi Y, Amaral DG (2003) Macaque monkey retrosplenial cortex: II. Cortical afferents. J Comp Neurol 466:48–79. 10.1002/cne.10883 14515240

[B40] Koh MT, Rosenzweig-Lipson S, Gallagher M (2013) Selective GABA(A) α5 positive allosteric modulators improve cognitive function in aged rats with memory impairment. Neuropharmacology 64:145–152. 10.1016/j.neuropharm.2012.06.023 22732440 PMC3445657

[B41] Kolarik BS, Baer T, Shahlaie K, Yonelinas AP, Ekstrom AD (2018) Close but no cigar: spatial precision deficits following medial temporal lobe lesions provide novel insight into theoretical models of navigation and memory. Hippocampus 28:31–41. 10.1002/hipo.22801 28888032 PMC5747326

[B42] Kolarik BS, Shahlaie K, Hassan AS, Borders AA, Kaufman KC, Gurkoff G, Yonelinas AP, Ekstrom AD (2016) Impairments in precision, rather than spatial strategy, characterize performance on the virtual Morris water maze: a case study. Neuropsychologia 80:90–101. 10.1016/j.neuropsychologia.2015.11.013 26593960 PMC4698252

[B43] Konishi K, Etchamendy N, Roy S, Marighetto A, Rajah N, Bohbot VD (2013) Decreased functional magnetic resonance imaging activity in the hippocampus in favor of the caudate nucleus in older adults tested in a virtual navigation task. Hippocampus 23:1005–1014. 10.1002/hipo.22181 23929534

[B44] Leal SL, Landau SM, Bell RK, Jagust WJ (2017) Hippocampal activation is associated with longitudinal amyloid accumulation and cognitive decline. Elife 6:e22978. 10.7554/eLife.22978 28177283 PMC5325620

[B45] Lester AW, Moffat SD, Wiener JM, Barnes CA, Wolbers T (2017) The aging navigational system. Neuron 95:1019–1035. 10.1016/j.neuron.2017.06.037 28858613 PMC5659315

[B46] Li B, Daunizeau J, Stephan KE, Penny WD, Hu D, Friston K (2011) Generalised filtering and stochastic DCM for fMRI. Neuroimage 58:442–457. 10.1016/j.neuroimage.2011.01.085 21310247

[B47] Luis CA, Keegan AP, Mullan M (2009) Cross validation of the Montreal Cognitive Assessment in community dwelling older adults residing in the Southeastern US. Int J Geriatr Psychiatry 24:197–201. 10.1002/gps.2101 18850670

[B48] Mao D, Neumann AR, Sun J, Bonin V, Mohajerani MH, McNaughton BL (2018) Hippocampus-dependent emergence of spatial sequence coding in retrosplenial cortex. Proc Natl Acad Sci USA 115:8015–8018. 10.1073/pnas.1803224115 30012620 PMC6077725

[B49] Miller AMP, Vedder LC, Law LM, Smith DM (2014) Cues, context, and long-term memory: the role of the retrosplenial cortex in spatial cognition. Front Hum Neurosci 8:1–15. 25140141 10.3389/fnhum.2014.00586 PMC4122222

[B50] Moffat SD, Elkins W, Resnick SM (2006) Age differences in the neural systems supporting human allocentric spatial navigation. Neurobiol Aging 27:965–972. 10.1016/j.neurobiolaging.2005.05.011 15982787

[B51] Morcom AM, Henson RNA (2018) Increased Prefrontal activity with aging reflects nonspecific neural responses rather than compensation. J Neurosci 38:7303–7313. 10.1523/JNEUROSCI.1701-17.2018 30037829 PMC6096047

[B52] Muffato V, Hilton C, Meneghetti C, De Beni R, Wiener JM (2019) Evidence for age-related deficits in object-location binding during place recognition. Hippocampus 29:971–979. 10.1002/hipo.23099 31070289

[B53] Nasreddine ZS, Phillips NA, Bédirian V, Charbonneau S, Whitehead V, Collin I, Cummings JL, Chertkow H (2005) The Montreal Cognitive Assessment, MoCA: a brief screening tool for mild cognitive impairment. J Am Geriatr Soc 53:695–699. 10.1111/j.1532-5415.2005.53221.x 15817019

[B54] Nilakantan AS, Bridge DJ, VanHaerents S, Voss JL (2018) Distinguishing the precision of spatial recollection from its success: evidence from healthy aging and unilateral mesial temporal lobe resection. Neuropsychologia 119:101–106. 10.1016/j.neuropsychologia.2018.07.035 30086364 PMC6191347

[B55] Oldfield RC (1971) The assessment and analysis of handedness: the Edinburgh inventory. Neuropsychologia 9:97–113. 10.1016/0028-3932(71)90067-4 5146491

[B56] Patai EZ, Javadi A-H, Ozubko JD, O'Callaghan A, Ji S, Robin J, Grady C, Winocur G, Rosenbaum RS, Moscovitch M, Spiers HJ (2019) Hippocampal and retrosplenial goal distance coding after long-term consolidation of a real-world environment. Cereb Cortex 29:2748–2758. 30916744 10.1093/cercor/bhz044 PMC6519689

[B57] Pedregosa F, Varoquaux G, Gramfort A, Michel V, Thirion B, Grisel O, Blondel M, Prettenhofer P, Weiss R, Dubourg V, Vanderplas J, Passos A, Cournapeau D, Brucher M, Perrot M, Duchesnay E (2011) Scikit-learn: machine learning in python. J Mach Learn Res 12:2825–2830.

[B58] Poppenk J, Evensmoen HR, Moscovitch M, Nadel L (2013) Long-axis specialization of the human hippocampus. Trends Cogn Sci 17:230–240. 10.1016/j.tics.2013.03.005 23597720

[B59] Reagh ZM, Noche JA, Tustison NJ, Delisle D, Murray EA, Yassa MA (2018) Functional imbalance of anterolateral entorhinal cortex and hippocampal dentate/CA3 underlies age-related object pattern separation deficits. Neuron 97:1187–1198. 10.1016/j.neuron.2018.01.039 29518359 PMC5937538

[B60] Robitsek J, Ratner MH, Stewart T, Eichenbaum H, Farb DH (2015) Combined administration of levetiracetam and valproic acid attenuates age-related hyperactivity of CA3 place cells, reduces place field area, and increases spatial information content in aged rat hippocampus. Hippocampus 25:1541–1555. 10.1002/hipo.22474 25941121 PMC4633399

[B61] Rosenbaum RS, Winocur G, Binns MA, Moscovitch M (2012) Remote spatial memory in aging: all is not lost. Front Ag Neurosci 4:25. 10.3389/fnagi.2012.00025 PMC3440628 22993506

[B62] Rugg MD, Morcom AM (2005) The relationship between brain activity, cognitive performance and aging: the case of memory. In: Cognitive neuroscience of aging: linking cognitive and cerebral aging (Cabeza R, Nyberg L, Park DC, eds), pp 132–154. New York: Oxford University Press.

[B63] Segen V, Avraamides MN, Slattery TJ, Wiener JM (2021) Age-related differences in visual encoding and response strategies contribute to spatial memory deficits. Memory & Cognition 49:249–264. 32869141 10.3758/s13421-020-01089-3 PMC7886755

[B64] Shine JP, Valdés-Herrera JP, Tempelmann C, Wolbers T (2019) Evidence for allocentric boundary and goal direction information in the human entorhinal cortex and subiculum. Nat Commun 10:4004. 10.1038/s41467-019-11802-9 31488828 PMC6728372

[B65] Smith AC, Wirth S, Suzuki WA, Brown EN (2007) Bayesian analysis of interleaved learning and response bias in behavioral experiments. J Neurophysiol 97:2516–2524. 10.1152/jn.00946.2006 17182907

[B66] Stan Development Team (2017) PyStan: the Python interface to Stan. 2.16.0.0. edition. Available at http://mc-stan.org.

[B67] Thomé A, Gray DT, Erickson CA, Lipa P, Barnes CA (2016) Memory impairment in aged primates is associated with region-specific network dysfunction. Mol Psychiatry 21:1257–1262. 10.1038/mp.2015.160 26503764 PMC4848213

[B68] Vehtari A, Gelman A, Gabry J (2017) Practical Bayesian model evaluation using leave-one-out cross-validation and WAIC. Stat Comput 27:1413–1432. 10.1007/s11222-016-9696-4

[B69] Vieweg P, Stangl M, Howard LR, Wolbers T (2015) Changes in pattern completion – a key mechanism to explain age-related recognition memory deficits? Cortex 64:343–351. 10.1016/j.cortex.2014.12.007 25597525 PMC4884644

[B70] Wang C, Chen X, Lee H, Deshmukh SS, Yoganarasimha D, Savelli F, Knierim JJ (2018) Egocentric coding of external items in the lateral entorhinal cortex. Science 362:945–949. 10.1126/science.aau4940 30467169 PMC6261310

[B71] Wiener JM, de Condappa O, Harris MA, Wolbers T (2013) Maladaptive bias for extrahippocampal navigation strategies in aging humans. J Neurosci 33:6012–6017. 10.1523/JNEUROSCI.0717-12.2013 23554482 PMC6618910

[B72] Wilson IA, Ikonen S, Gallagher M, Eichenbaum H, Tanila H (2005) Age-associated alterations of hippocampal place cells are subregion specific. J Neurosci 25:6877–6886. 10.1523/JNEUROSCI.1744-05.2005 16033897 PMC6725350

[B73] Wolbers T, Büchel C (2005) Dissociable retrosplenial and hippocampal contributions to successful formation of survey representations. J Neurosci 25:3333–3340. 10.1523/JNEUROSCI.4705-04.2005 15800188 PMC6724902

[B74] Yamamoto N, DeGirolamo G (2012) Differential effects of aging on spatial learning through exploratory navigation and map reading. Front Ag Neurosci 4:14. 10.3389/fnagi.2012.00014 PMC3372958 22701423

[B75] Yassa MA, Lacy JW, Stark SM, Albert MS, Gallagher M, Stark CEL (2011) Pattern separation deficits associated with increased hippocampal CA3 and dentate gyrus activity in nondemented older adults. Hippocampus 21:968–979. 10.1002/hipo.20808 20865732 PMC3010452

[B76] Zeidman P, Jafarian A, Seghier ML, Litvak V, Cagnan H, Price CJ, Friston KJ (2019a) A guide to group effective connectivity analysis, part 2: second level analysis with PEB. Neuroimage 200:12–25. 10.1016/j.neuroimage.2019.06.032 31226492 PMC6711451

[B77] Zeidman P, Jafarian A, Corbin N, Seghier ML, Razi A, Price CJ, Friston KJ (2019b) A guide to group effective connectivity analysis, part 1: first level analysis with DCM for fMRI. Neuroimage 200:174–190. 10.1016/j.neuroimage.2019.06.031 31226497 PMC6711459

[B78] Zeidman P, Maguire EA (2016) Anterior hippocampus: the anatomy of perception, imagination and episodic memory. Nat Rev Neurosci 17:173–182. 10.1038/nrn.2015.24 26865022 PMC5358751

