# Supplementary material for: Increased Hippocampal Excitability and Altered Learning Dynamics Mediate Cognitive Mapping Deficits in Human Aging
Source: J Neurosci. 2021 Apr 7;41(14):3204–21. doi: 10.1523/JNEUROSCI.0528-20.2021 (PMC8026345; doi:10.1523/JNEUROSCI.0528-20.2021)
Supplement: Extended Data Figure 2-1 — Stan code of the Bayesian state-space model. Download Figure 2-1, DOCX file. [file ns-JN-RM-0528-20-s02.docx]

data {

int<lower=0> nblocks;

int<lower=0> ntrials;

matrix[nblocks, ntrials] y; // the data

int<lower=0, upper=1> fit_model; // whether to fit data or not

// vars controlling the priors

real theta_loc;

real theta_scale;

real mulog1_loc;

real mulog1_scale;

real mulog_scale;

real sigma_loc;

real sigma_scale;

}

parameters {

real<lower=0> theta; // theta is also *regularizing* the random effects

vector<lower=0,upper=log(180)>[nblocks] mulog;

matrix<lower=0>[nblocks, ntrials] eta;

vector<lower=0>[nblocks] sigma;

}

model {

theta ~ normal(theta_loc, theta_scale);

mulog[1] ~ normal(mulog1_loc, mulog1_scale);

for (b in 2:nblocks) {

mulog[b] ~ normal(mulog[b - 1], mulog_scale);

}

for (b in 1:nblocks) {

sigma[b] ~ normal(sigma_loc, sigma_scale);

}

if (fit_model == 1) {

for (b in 1:nblocks) {

for (t in 1:ntrials) {

if (y[b, t] >= 0) {

eta[b, t] ~ normal(mulog[b], theta);

y[b, t] ~ lognormal(eta[b, t], sigma[b]);

}

else {

eta[b, t] ~ normal(log(mean(y[b, :])), 1);

}

}

}

}

}

generated quantities{

vector<lower=0>[nblocks] expected_val;

vector<lower=0>[nblocks] variance_val;

matrix[nblocks, ntrials] log_lik; // log likelihood

for (b in 1:nblocks) {

expected_val[b] = exp(mulog[b] + ((sigma[b] ^ 2) / 2));

variance_val[b] = exp((2 * mulog[b]) + (sigma[b] ^ 2)) * (exp(sigma[b] ^ 2) - 1);

for (t in 1:ntrials) {

if (y[b, t] >= 0) {

log_lik[b, t] = lognormal_lpdf(y[b, t] | eta[b, t], sigma[b]);

}

}

}

}
