# Supplementary figures and images for: Increased Hippocampal Excitability and Altered Learning Dynamics Mediate Cognitive Mapping Deficits in Human Aging
Source: J Neurosci. 2021 Apr 7;41(14):3204–21. doi: 10.1523/JNEUROSCI.0528-20.2021 (PMC8026345; doi:10.1523/JNEUROSCI.0528-20.2021)

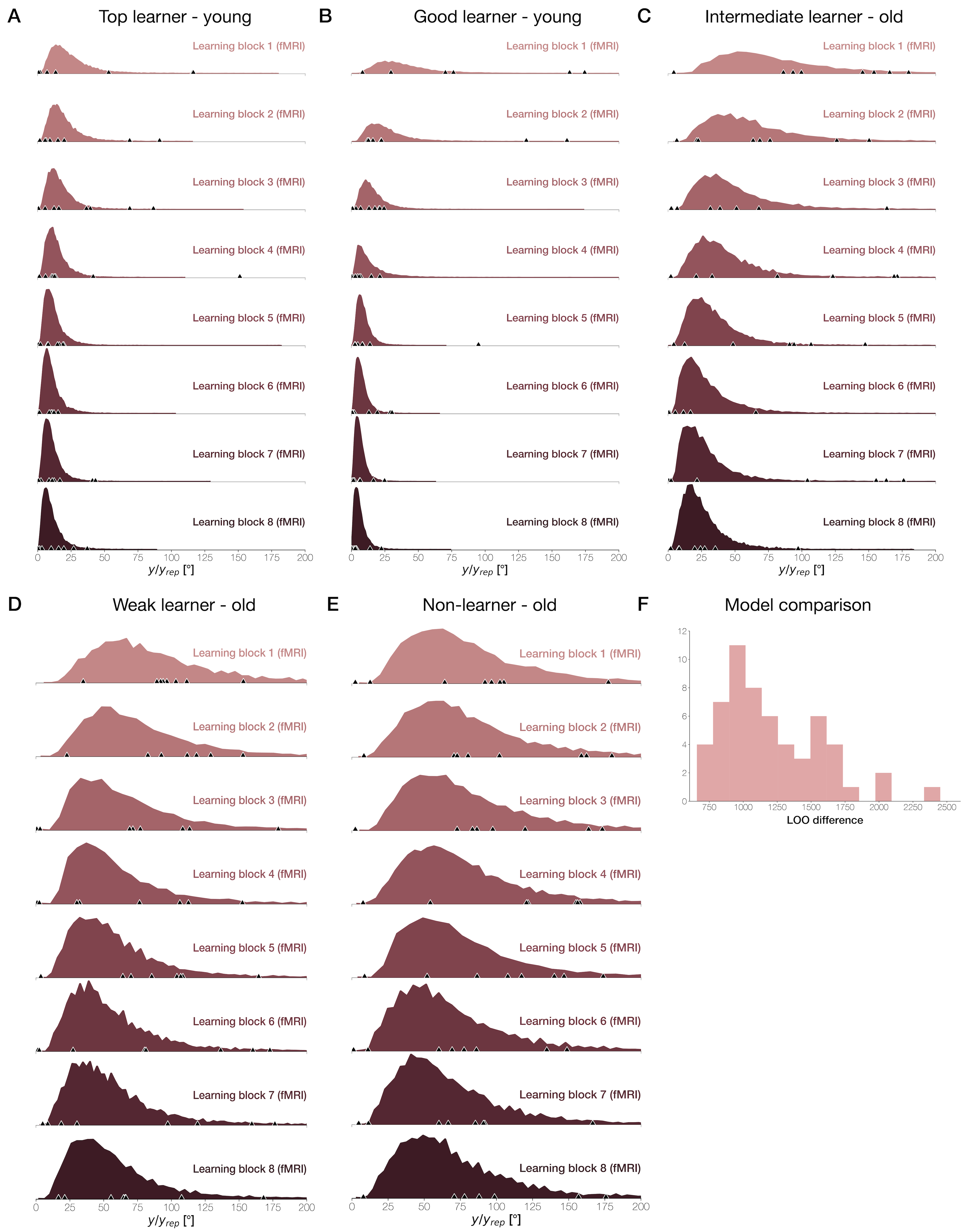

Supplement: Extended Data Figure 2-2 — Results of the posterior predictive checks of the Bayesian state-space model for representative individuals from each learning subgroup (A, top learner young to E, non-learner old; see Performance clustering section; the posterior predictive samples distribution, yrep, plotted together with the observed data points, y, per learning block) and (F) histogram of the individuals' LOO differences for the comparison of the Bayesian state-space model incorporating the effects of the responses, η, to an alternative model that estimated the individuals' learning state trial-wise. More positive values indicate a better fit of the first model. Download Figure 2-2, TIF file. [file ns-JN-RM-0528-20-s03.tif]

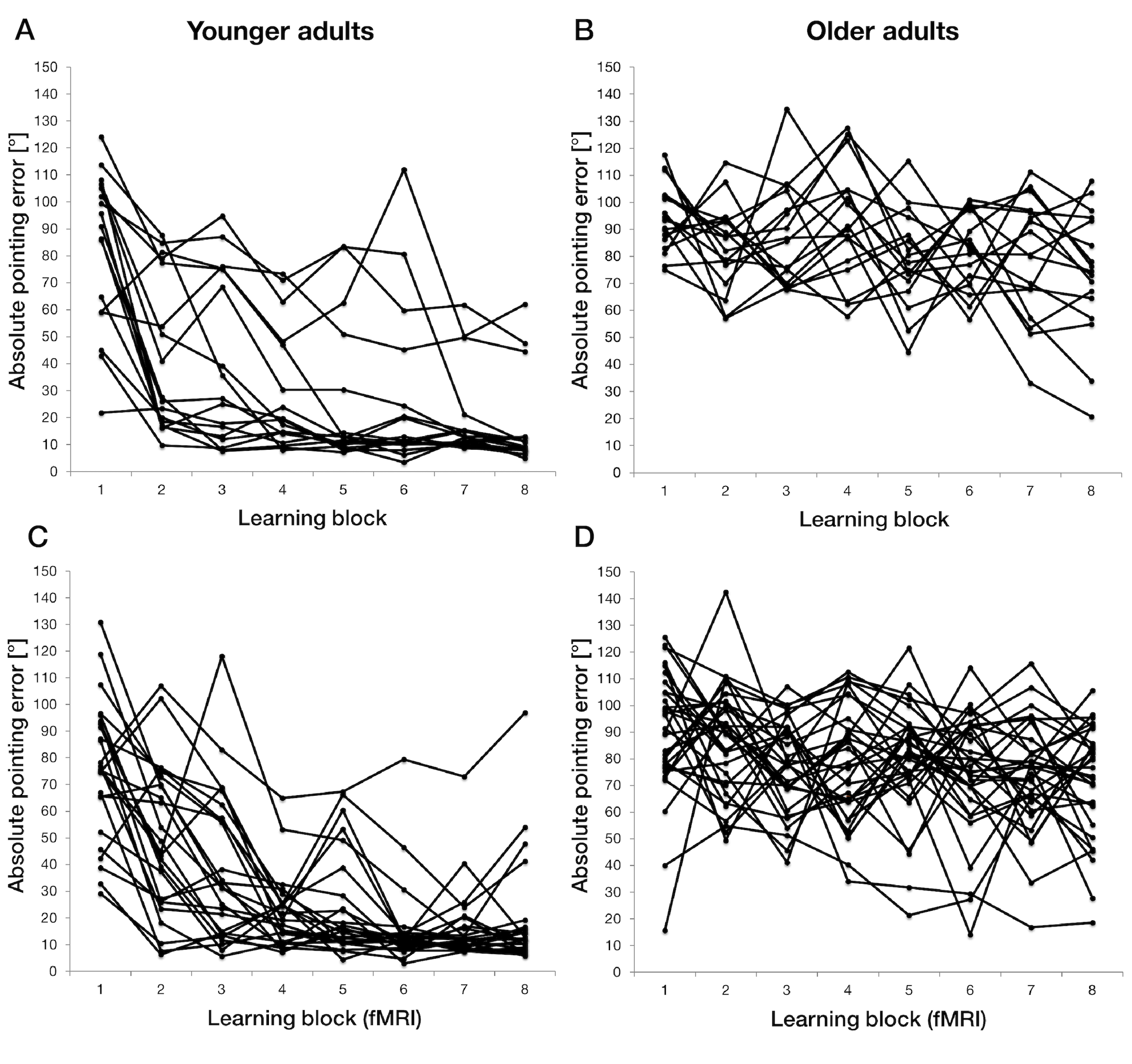

Supplement: Extended Data Figure 3-1 — Average absolute pointing errors per learning block for each participant in (A) the younger and (B) the older age group in the behavioral experiment and for each participant in (C) the younger and (D) the older age group in the fMRI experiment. Download Figure 3-1, TIF file. [file ns-JN-RM-0528-20-s04.tif]

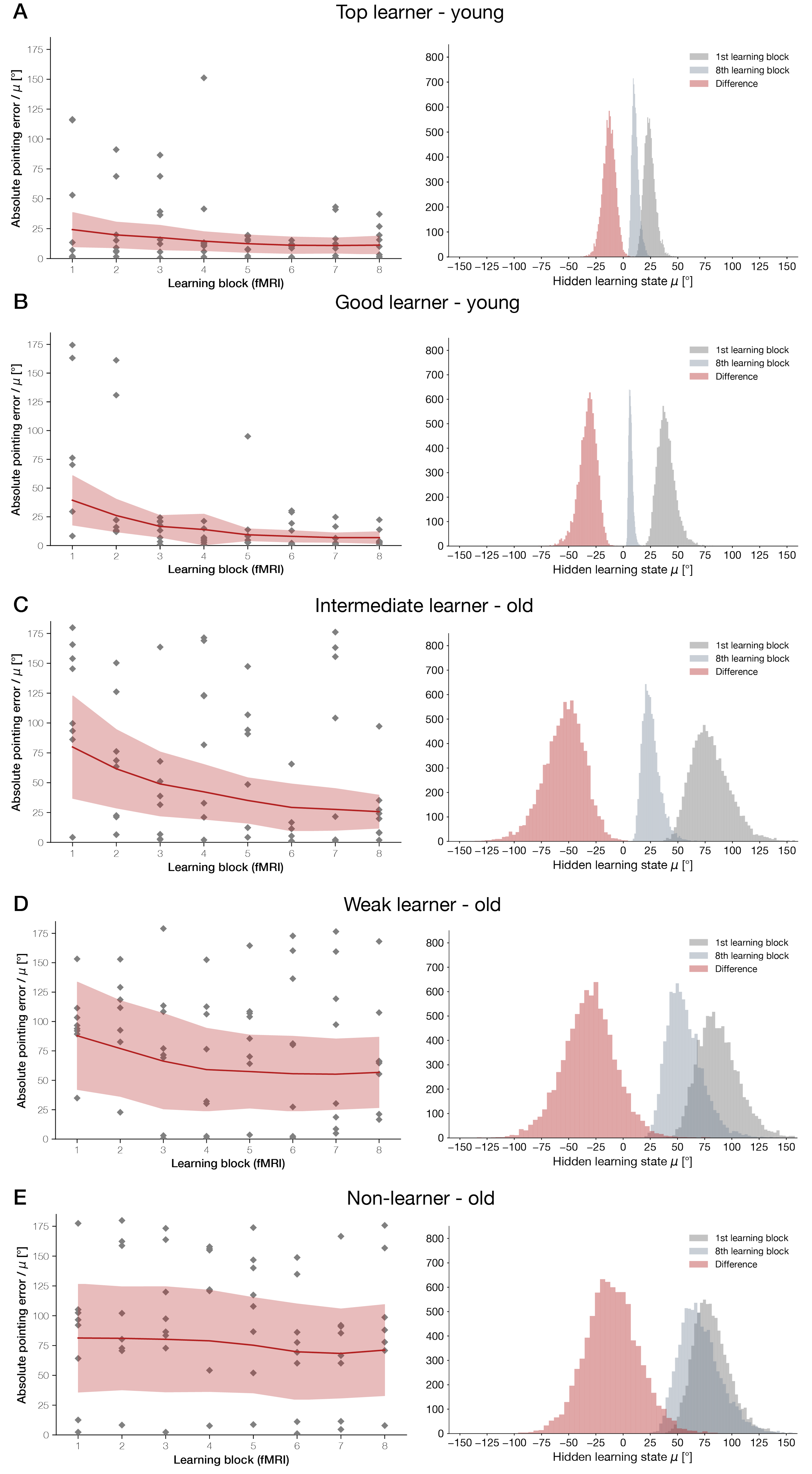

Supplement: Extended Data Figure 4-1 — Definition of learning subgroups. Hidden learning states (including SD) and trial-wise performance data per learning block (left) and the latent state distributions of the last and first learning block plotted together with the difference distribution (right) from representative individuals from each learning subgroup in the fMRI experiment. Download Figure 4-1, TIF file. [file ns-JN-RM-0528-20-s05.tif]

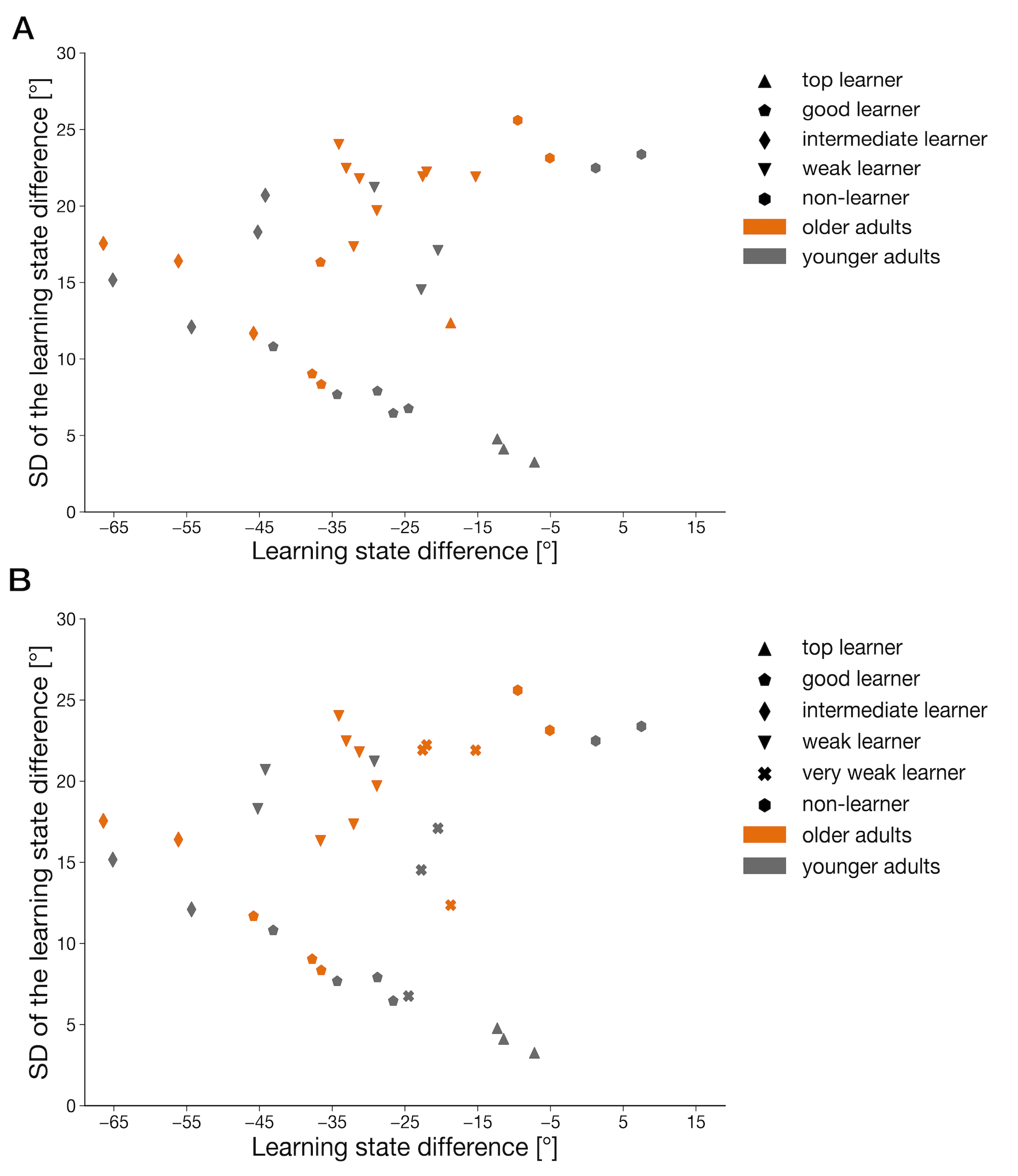

Supplement: Extended Data Figure 4-2 — Learning subgroups in the behavioral experiment as identified by a K-means clustering algorithm based on the individuals' overall amount of learning and its SD, as determined by the difference of the latent state distributions of the last and first learning block. Results are shown for (A) five and (B) six learning clusters that yielded similar silhouette scores (respective mean silhouette scores per tested cluster number: 3: 0.232, 4: 0.293, 5: 0.400, 6: 0.404, 7: 0.370). Download Figure 4-2, TIF file. [file ns-JN-RM-0528-20-s06.tif]
