# Supplementary material for: Increased Hippocampal Excitability and Altered Learning Dynamics Mediate Cognitive Mapping Deficits in Human Aging
Source: J Neurosci. 2021 Apr 7;41(14):3204–21. doi: 10.1523/JNEUROSCI.0528-20.2021 (PMC8026345; doi:10.1523/JNEUROSCI.0528-20.2021)
Supplement: Extended Data Figure 4-3 — Key demographics of the learning subgroups within each age group in the fMRI experiment. Download Figure 4-3, DOCX file. [file ns-JN-RM-0528-20-s07.docx]

|  |  | n | Sex | Age | MoCA score |
| --- | --- | --- | --- | --- | --- |
| 1. Younger adults | | | | | |
|  | Top | 7 | 3 female | 24.6 ± 0.98 | -- |
|  | Good | 14 | 6 female | 22.9 ± 2.41 | -- |
|  | Intermediate | 3 | 3 female | 23.0 ± 3.00 | -- |
|  | Non-learner | 1 | 1 female | 24 | -- |
| 1. Older adults | | | | | |
|  | Top | 2 | 1 female | 68.5 ± 6.36 | 27.5 ± 3.54 |
|  | Intermediate | 6 | 2 female | 65.5 ± 5.21 | 28.2 ± 2.23 |
|  | Weak | 12 | 6 female | 67.8 ± 5.32 | 27.7 ± 2.23 |
|  | Non-learner | 12 | 8 female | 67.6 ± 4.27 | 27.3 ± 1.37 |
