# Supplementary material for: Increased Hippocampal Excitability and Altered Learning Dynamics Mediate Cognitive Mapping Deficits in Human Aging
Source: J Neurosci. 2021 Apr 7;41(14):3204–21. doi: 10.1523/JNEUROSCI.0528-20.2021 (PMC8026345; doi:10.1523/JNEUROSCI.0528-20.2021)
Supplement: Extended Data Table 1-1 — Spatial coordinates of the local maxima in the whole-brain fMRI analyses on age-related differences in neural activation patterns (p < 0.05, whole-brain FWE-corrected). Download Table 1-1, DOCX file. [file ns-JN-RM-0528-20-s01.docx]

|  | Brain region | Cluster size | MNI coordinate | | | Z-score |
| --- | --- | --- | --- | --- | --- | --- |
|  | |  | x | y | z |  |
| 1. Navigation vs. control across the whole sample | | | | | | |
|  | L Parieto-occipital sulcus | 5009 | -18 | -60 | 21 | Inf |
|  | R Retrosplenial cortex |  | 15 | -54 | 21 | Inf |
|  | L Angular gyrus |  | -33 | -81 | 34 | Inf |
|  | R Premotor cortex | 2594 | 33 | 6 | 57 | Inf |
|  | L Premotor cortex |  | -30 | 27 | 1 | 7.54 |
|  | Supplementary motor area |  | 0 | 15 | 47 | 7.18 |
|  | R Cerebellum | 204 | 30 | -63 | -32 | 6.58 |
|  |  |  | 39 | -69 | -29 | 6.42 |
|  | L Inferior frontal gyrus | 201 | -36 | 51 | 4 | 4.91 |
|  | L Middle frontal gyrus |  | -36 | 54 | 18 | 4.76 |
| 1. Reduced activity in older compared to younger adults during navigation vs. control | | | | | | |
|  | L Cerebellum | 69 | -36 | -45 | -35 | 4.91 |
|  | L Precuneus | 245 | -9 | -78 | 54 | 4.80 |
|  | R Precuneus |  | 12 | -69 | 57 | 4.45 |
|  | L Supramarginal gyrus | 82 | -48 | -42 | 57 | 4.08 |
|  | R Superior parietal lobe | 70 | 39 | -42 | 51 | 4.07 |
|  | R Supramarginal gyrus | 94 | 57 | -30 | 41 | 4.01 |
|  | R Postcentral gyrus |  | 66 | -18 | 37 | 3.30 |
|  | R Insula | 73 | 30 | 18 | 8 | 3.82 |
|  | R Premotor cortex |  | 48 | 6 | 14 | 3.66 |
| 1. Increased activity in older compared to younger adults during navigation vs. control | | | | | | |
|  | R Superior frontal gyrus | 2292 | 3 | 60 | 37 | 6.06 |
|  | L Ventromedial prefrontal cortex |  | -3 | 63 | 18 | 5.56 |
|  | L Orbitofrontal cortex | 380 | -36 | 33 | -12 | 6.03 |
|  | L Inferior frontal gyrus |  | -51 | 24 | -9 | 4.54 |
|  | R Middle temporal gyrus | 471 | 69 | -18 | -15 | 5.81 |
|  |  |  | 60 | -9 | -29 | 4.84 |
|  | L Cerebellum | 525 | -42 | -78 | -38 | 5.71 |
|  | L Visual cortex (extrastriate area) |  | -27 | -78 | -9 | 4.69 |
|  | L Angular gyrus | 368 | -54 | -66 | 28 | 5.38 |
|  | R Orbitofrontal gyrus | 232 | 30 | 33 | -12 | 5.32 |
|  | R Inferior frontal gyrus |  | 42 | 36 | -15 | 4.68 |
|  | R Visual cortex (extrastriate area) | 387 | 27 | -84 | -15 | 5.13 |
|  | R Cerebellum |  | 15 | -87 | -32 | 4.40 |
|  | R Angular gyrus | 245 | 45 | -57 | 31 | 4.99 |
|  |  |  | 54 | -66 | 47 | 4.82 |
|  | L Superior temporal sulcus | 487 | -63 | -39 | 1 | 4.84 |
|  |  |  | -57 | -3 | -15 | 4.35 |
|  | L Hippocampus | 82 | -21 | -18 | -12 | 4.76 |
|  |  |  | -30 | -15 | -22 | 4.21 |
|  | Posterior cingulate cortex | 192 | 0 | -60 | 34 | 4.57 |
|  | L Posterior cingulate cortex |  | -6 | -57 | 21 | 4.32 |
|  | L Premotor cortex | 354 | -39 | 12 | 24 | 4.55 |
|  | L Middle frontal gyrus |  | -42 | 21 | 47 | 4.26 |
|  | R Precentral gyrus | 120 | 33 | -15 | 28 | 4.23 |
|  |  |  | 36 | -15 | 41 | 4.00 |
| 1. Retrieval vs. encoding across the whole sample | | | | | | |
|  | R Caudate | 8550 | 12 | 12 | -2 | Inf |
|  | L Premotor Cortex |  | -48 | 0 | 34 | Inf |
|  | L Fusiform Gyrus/Inferior temporal gyrus |  | -42 | -63 | -12 | Inf |
|  | L Parieto-occipital sulcus |  | -15 | -72 | 24 | 7.81 |
|  | L Lingual Gyrus |  | -21 | -54 | -2 | 7.79 |
|  | R Cerebellum | 241 | 6 | -69 | -22 | 5.73 |
| 1. Encoding vs. retrieval across the whole sample | | | | | | |
|  | L Visual cortex (extrastriate area) | 9128 | -6 | -87 | -9 | Inf |
|  | R Visual cortex (extrastriate area) |  | 12 | -90 | -9 | Inf |
|  | R Superior frontal gyrus | 387 | 30 | 39 | 51 | 4.89 |
|  |  |  | 39 | 30 | 41 | 4.78 |
|  | L Superior frontal gyrus | 118 | -36 | 30 | 51 | 4.78 |
|  |  |  | -24 | 27 | 51 | 4.00 |
|  | L Superior frontal gyrus | 101 | -18 | 57 | 31 | 3.42 |
|  |  |  | -9 | 54 | 31 | 3.41 |
|  | R Anterior cingulate cortex | 86 | 3 | 21 | 24 | 4.15 |
| 1. Reduced activity in older compared to younger adults during retrieval vs. encoding | | | | | | |
|  | R Fusiform gyrus | 144 | 27 | -63 | -12 | 4.56 |
|  |  |  | 36 | -78 | -15 | 4.17 |
|  | L Brain stem | 92 | -3 | -45 | -48 | 4.17 |
|  |  |  | 0 | -39 | -38 | 3.81 |
| 1. Increased activity in older compared to younger adults during retrieval vs. encoding | | | | | | |
|  | R Supramarginal gyrus | 2863 | 63 | -24 | 24 | 6.23 |
|  | R Inferior frontal gyrus |  | 57 | 15 | -2 | 5.16 |
|  | L Postcentral gyrus | 196 | -24 | -39 | 67 | 5.61 |
|  | R Posterior cingulate cortex | 438 | 6 | -27 | 41 | 5.56 |
|  |  |  | 9 | -51 | 34 | 4.07 |
|  | L Middle temporal gyrus | 2422 | -54 | -54 | -2 | 5.44 |
|  | L Insula |  | -39 | -15 | -5 | 5.43 |
|  | L Superior temporal gyrus |  | -66 | -39 | 24 | 5.40 |
|  | R Rostral prefrontal cortex | 1180 | 9 | 54 | -2 | 4.59 |
|  | L Anterior cingulate cortex |  | -3 | 27 | 18 | 4.50 |
|  | R Superior frontal gyrus |  | 24 | 51 | 34 | 4.42 |
|  | L Cerebellum | 220 | -27 | -84 | -32 | 4.49 |
|  |  |  | -39 | -72 | -42 | 4.22 |
|  | L Superior frontal gyrus | 99 | -27 | 45 | 24 | 4.07 |
|  | L Middle frontal gyrus |  | -33 | 57 | 21 | 3.62 |
| 1. Age-group differences in learning-related  activity decreases | | | | | | |
|  | L Ventromedial prefrontal cortex | 202 | -12 | 54 | -2 | 4.04 |
|  |  |  | -3 | 42 | -12 | 4.02 |
|  | R Superior frontal gyrus | 71 | 0 | 42 | 37 | 4.02 |
|  |  |  | 9 | 45 | 41 | 3.82 |
| 1. Age-group differences in learning-related  activity increases | | | | | | |
|  | L Parieto-occipital sulcus | 2257 | -9 | -66 | 24 | 5.93 |
|  |  |  | -27 | -69 | 51 | 5.52 |
|  | R Retrosplenial cortex |  | 9 | -57 | 4 | 4.82 |
|  | Supplementary motor area | 203 | 3 | 3 | 61 | 5.45 |
|  |  |  | -9 | 6 | 41 | 3.81 |
|  | R Premotor cortex | 125 | 30 | 0 | 51 | 4.81 |
|  |  |  | 27 | -3 | 67 | 4.44 |
|  | L Premotor cortex | 138 | -24 | 3 | 57 | 4.57 |
|  |  |  | -30 | -6 | 57 | 4.49 |
|  | Visual cortex (striate area) | 257 | -6 | -102 | -2 | 4.54 |
|  |  |  | 12 | -102 | 11 | 3.96 |
