# Supplementary material for: Increased Hippocampal Excitability and Altered Learning Dynamics Mediate Cognitive Mapping Deficits in Human Aging
Source: J Neurosci. 2021 Apr 7;41(14):3204–21. doi: 10.1523/JNEUROSCI.0528-20.2021 (PMC8026345; doi:10.1523/JNEUROSCI.0528-20.2021)
Supplement: Extended Data Figure 6-1 — Spatial coordinates of the local maxima in the fMRI analyses on inter-individual differences in neural activation patterns across learning blocks within older adults (p < 0.05, FWE-corrected). Download Figure 6-1, DOCX file. [file ns-JN-RM-0528-20-s08.docx]

|  | Brain region | Cluster size | MNI coordinate | | | Z-score |
| --- | --- | --- | --- | --- | --- | --- |
|  | |  | x | y | z |  |
|  | L Cerebellum | 3379 | -27 | -57 | -48 | 6.15 |
|  | R Thalamus |  | 6 | -15 | 18 | 6.12 |
|  | L Visual Cortex |  | -9 | -87 | 31 | 5.76 |
|  | L Inferior Temporal Gyrus | 199 | -48 | -12 | -29 | 5.88 |
|  | L Superior Temporal Gyrus |  | -42 | -15 | -12 | 4.39 |
|  | R Hippocampus | 110 | 39 | -9 | -25 | 5.78 |
|  | R Parahippocampus |  | 36 | -6 | -32 | 5.55 |
|  | L Precuneus | 134 | -12 | -51 | 47 | 5.28 |
|  |  |  | -6 | -54 | 51 | 4.88 |
|  | L Paracentral Lobule | 941 | -6 | -33 | 57 | 5.13 |
|  | R Precentral Gyrus |  | 27 | -24 | 54 | 5.12 |
|  | L Precentral Gyrus |  | -36 | -12 | 47 | 5.07 |
|  | R Middle Frontal Gyrus | 64 | 36 | 15 | 34 | 4.80 |
|  |  |  | 39 | 18 | 31 | 4.50 |
|  | R Middle Frontal Gyrus | 134 | 18 | 6 | 54 | 4.78 |
|  | R Precentral Gyrus |  | 45 | -6 | 51 | 4.52 |
|  | L Middle Temporal Gyrus | 76 | -60 | -42 | -5 | 4.07 |
|  |  |  | -60 | -30 | -2 | 3.99 |
